# Liquisolid Technology Improves and Reduces Source-Dependent Variability in Andrographolide Dissolution from *Andrographis paniculata* Extracts

**DOI:** 10.3390/pharmaceutics18080976

**Published:** 2026-08-08

**Authors:** Peera Tabboon, Ekapol Limpongsa, Sarunya Tuntiyasawasdikul, Tanyarat Yodthong, Watchara Kanjanakawinkul, Napaphak Jaipakdee

**Affiliations:** 1 Faculty of Pharmaceutical Sciences, Khon Kaen University, Khon Kaen 40002, Thailand; peerata@kku.ac.th (P.T.); sarunyat@kku.ac.th (S.T.); tanyarat.y@kkumail.com (T.Y.); 2 Center for Research and Development of Herbal Health Products, Khon Kaen University, Khon Kaen 40002, Thailand; 3 College of Pharmacy, Rangsit University, Pathum Thani 12000, Thailand; ekapol.l@rsu.ac.th; 4 Chulabhorn Royal Pharmaceutical Manufacturing Facilities, Chulabhorn Royal Academy, Chon Buri 20180, Thailand; watchara.kan@cra.ac.th

**Keywords:** andrographolide, diterpene lactone, dissolution enhancement, reduction of source-dependent dissolution variation, liquisolid technology, powder solution technology

## Abstract

**Background/Objectives**: Andrographolide (AG), the primary diterpene of *Andrographis paniculata*, is classified as a BCS Class II substance, with its dissolution known to be restricted and inconsistent across various Andrographis extracts and formulations. This study aimed to implement liquisolid (LS) technology to enhance and reduce source-dependent variability in the dissolution of AG from *Andrographis paniculata* extracts (APEs). **Methods**: Ethanolic APEs from three distinct sources in Thailand were obtained using Soxhlet extraction. The AG content in the APEs varied between 14.9 and 24.1%. LS powders of APEs and pure AG were fabricated using microcrystalline cellulose and colloidal silicon dioxide as the carriers and coating materials. Their FTIR, PXRD, and SEM characteristics, flowability, and AG content were investigated. **Results**: All LS formulations exhibited good flowability and assay consistency. FTIR studies suggested intermolecular interactions between the α,β-unsaturated lactone ring of AG and the liquid vehicle. PXRD and SEM studies revealed the non-crystalline state of AG in LS powder, regardless of the AG source. All LS powders, regardless of the AG source, exhibited improved and similar dissolution profiles, with a similarity factor >51 under both sink and non-sink conditions. Preliminary stability assessments demonstrated that all formulated LS powders exhibited no marked physical changes over three months, regardless of the storage conditions. The percentage of AG remaining was significantly affected by storage temperature. Only LS powders stored under refrigerated conditions maintained dissolution patterns similar to those of fresh samples. **Conclusions**: This study offers foundational insights into LS strategies aimed at improving and reducing the source-dependent variability of herbal-extract powders.

## 1. Introduction

*Andrographis paniculata* (Burm. f.) Wall. ex Nees (*A. paniculata*) is a medicinal herb in the Acanthaceae family that has been utilized in many traditional medicines in Asian countries for treating numerous health concerns [1,2,3]. *A. paniculata* crude extract exhibits an array of pharmacological effects [1,2,4,5,6,7,8]. Phytochemical examination of *A. paniculata* has identified up to 55 varieties of *ent*-labdane diterpenoids, 30 forms of flavonoids, and various polyphenols [3,5,9]. Among these, andrographolide (AG) is the principal diterpene of *A. paniculata* and is the key constituent responsible for its pharmacological effects. Other significant bioactive labdane diterpene lactones include 14-deoxy-11,12-didehydroandrographolide (DDAG), neoandrographolide, and 14-deoxyandrographolide [2,4,9,10]. The estimated proportion of diterpene lactone compounds in the aerial portions of *A. paniculata* may reach approximately 35%, calculated based on the compounds isolated from the resultant extracts [2].

Currently, commercially available preparations of *A. paniculata* include various solid dosage forms (tablets, capsules, and granules) and liquid formulations (syrups, drops, or tinctures) derived from dried powdered samples, unprocessed aerial parts, or extracts [11,12]. However, these preparations have not yet achieved a level of standardization that ensures their anticipated efficacy [3]. Previous studies have shown that AG compounds exhibit poor water solubility and limited dissolution properties [3,12,13]. Compared to the other three diterpene lactones, AG dissolved in all the dissolution media at a lower percentage [14]. Additionally, significant variations in the content and dissolution behaviors of AG and other diterpene lactones (DDAG, neoandrographolide, and 14-deoxyandrographolide) from different sources of *A. paniculata* crude extract (Andrographis extract, APE) have been demonstrated [10,14,15]. The restricted and variable dissolution characteristics of diterpene lactones may lead to varying bioavailability, posing a considerable challenge to achieving comparable pharmacological effects and clinical applicability [3,14]. Suitable pharmaceutical dissolution enhancement strategies are required to improve and reduce source-dependent variability in the dissolution behavior of diterpene lactones in *A. paniculata* preparations.

Numerous studies have demonstrated that dissolution enhancement technology can significantly improve the oral absorption and bioavailability of BCS Class II drugs, including AG compounds, which exhibit dissolution-limited absorption characteristics [13,16,17]. Various pharmaceutical technologies have been effectively employed to develop dissolution-enhancing formulations of pure AG (pAG) and APEs, yielding different levels of bioavailability enhancement for AG. These technologies include nanotechnology-based delivery systems [18,19,20,21,22,23], soluble complex formation [24], amorphous solid dispersions [25,26,27], and liquisolid (LS) technology [28,29]. To date, no study has investigated the application of dissolution enhancement technology to reduce the variation in the dissolution behavior of AG compounds from various sources.

A straightforward yet successful approach to improve the dissolution of BCS Class II compounds is the use of the LS technique. The concept of “LS” refers to a method in which an active BCS class II compound in a liquid medication form is converted into a dry, free-flowing powder. The resulting powder, called an LS powder, is suitable for tablet or capsule filling. Liquid medications typically consist of a solution or suspension of a BCS class II compound that is either dissolved or dispersed in a liquid vehicle. Liquid medications are transformed into dry, free-flowing powders by integrating porous excipients such as carriers and coating materials [16,30]. LS technology has been documented to enhance the dissolution behavior of several drugs and phytopharmaceuticals [31,32,33,34,35,36,37,38]. These enhanced characteristics may ultimately increase the oral bioavailability of these compounds [28,31,39,40,41,42]. Recent reports have demonstrated that LS systems based on starch [28] and Neusilin [29] carriers effectively improve the in vivo bioavailability of AG compounds.

The utilization of the LS approach to improve and reduce the source-dependent variability of phytopharmaceuticals from diverse sources of crude extracts is of great significance. The dry, free-flowing product, simplicity of preparation, and scalability of LS technology make it suitable for producing crude-extract powders. The present investigation aimed to implement LS technology to enhance and reduce the variability in the dissolution of AG from different APE sources. An LS powder of pAG was produced and examined for comparative analysis. Three different APEs derived from three different sources were obtained using the same extraction protocols. LS systems in the form of LS powders of APEs were fabricated using microcrystalline cellulose (MCC) as the carrier and colloidal silicon dioxide (CSD) as the coating material. The MCC–CSD system was selected owing to it being a long-established and commercially accessible option at an affordable cost. Additionally, this carrier-coating material has not yet been applied to prepare LS systems of pAG and APEs. A liquid solvent with superior solubility for AG compounds was examined and used as a liquid vehicle in the designed LS systems. Their AG content; SEM, PXRD, and FTIR characteristics; dissolution; and stability were systematically examined. Notably, this study exclusively focused on the characteristics of AG molecules.

## 2. Materials and Methods

### 2.1. Materials

#### 2.1.1. Plant Materials

The dried leaves of *Andrographis paniculata* (Burm. f.) Wall. ex Nees (*A. paniculata*) were procured as three independent commercial batches from local medicinal community enterprises in Thailand, each located in a different region: source 1 is a community enterprise of local herbal growers and processors in Ban Non Tae, Village No. 10, Sam Chai District, Kalasin Province (AP–S1), which completed its harvest in June 2023; source 2 is the Ban Dong Bang Herbal Community, Dong Khilek Subdistrict of Mueang District, Prachinburi Province (AP–S2), which completed its harvest in March 2023; and source 3 is the Diamond Phu Phan Sacha Inchi Product Group, a community enterprise located in Phu Phan District, Sakon Nakhon Province (AP–S3), which conducted their harvest in March 2023. The specimens were verified by a botanist. The related herbarium collections (HB 200/66–HB 202/66) were maintained at the Center for Research and Development of Herbal Health Products, Khon Kaen University (Thailand). Dried leaves of *A. paniculata* were ground into a fine powder and preserved in an airtight container at a controlled temperature of 20–25 °C for future use. The AG content contained in dried leaves of AP–S1, AP–S2, and AP–S3 was determined to be 6.51 ± 0.40%, 4.14 ± 0.51%, and 4.02 ± 0.24%, respectively. The DDAG content was 0.59 ± 0.01%, 0.16 ± 0.02%, and 3.26 ± 0.22% for AP–S1, AP–S2, and AP–S3, respectively.

#### 2.1.2. Chemicals and Solvents

Pure andrographolide (pAG, >98% purity) and 14-deoxy-11,12-didehydroandrographolide (DDAG, >98% purity) were obtained from Weifang Wehibest Supply Chain Co., Ltd. (Shandong, China). The reference AG standard was purchased from the Bureau of Drug and Narcotic Control, Department of Medical Sciences, Ministry of Public Health (Nonthaburi, Thailand). N-Methyl-2-pyrrolidone (NMP) was obtained from QRëC (Auckland, New Zealand). Caprylocaproyl macrogolglycerides (Labrasol^®^ ALF, Gattefossé SAS, Saint-Priest, France) were procured as complimentary samples from Rama Production Co., Ltd. (Bangkok, Thailand). Polyoxyl 35 castor oil (Kolliphor^®^ EL, BASF SE (Ludwigshafen, Germany)) was generously provided by BASF VITA Company Limited (Bangkok, Thailand). Transcutol (high-purity (>99.9%) pharmaceutical-grade Transcutol^®^ HP, Gattefossé SAS, Saint-Priest, France) was purchased from Rama Production Co., Ltd. (Bangkok, Thailand). Sodium dodecyl sulfate (SDS) was obtained from Loba Chemie Pvt. Ltd. (Mumbai, India). Colloidal silicon dioxide (CSD, Aerosil^®^ 200) was supplied by Maxway Co., Ltd. (Bangkok, Thailand). Microcrystalline cellulose (MCC, Avicel^®^ PH102) was obtained from Onimax Co., Ltd. (Bangkok, Thailand). Ethyl alcohol (EtOH, L Pure^®^ 99.8) was supplied by the Liquor Distillery Organization (Chachoengsao, Thailand). Polysorbate 20 (P20) was purchased from AppliChem GmbH (Darmstadt, Germany). Hydrochloric acid (HCl) was supplied by RCI Labscan Co., Ltd. (Bangkok, Thailand). Methanol and acetonitrile (HPLC grade) were obtained from Fisher Scientific (Loughborough, UK). All other substances were used as received without further purification.

### 2.2. APE Preparations

APEs derived from three different sources of *A. paniculata* leaves (APE–S1, APE–S2, and APE–S3) were prepared using the Soxhlet extraction method. Specifically, 500 g of *A. paniculata* leaf powder was placed in a thimble and subjected to Soxhlet extraction using absolute EtOH as the extraction solvent at a 1:6 ratio. The extraction process was conducted in a 5 L Soxhlet extractor with an electric heating mantle (LabHeat, type KM-ME, Hamburg, Germany) at 60 °C for 5 h. Following extraction, the *A. paniculata* powder residue was filtered using a thin cloth and Whatman filter paper (No. 1). The resulting mixture was then subjected to vacuum rotary evaporation using a Heidolph Hei-VAP rotary evaporator (Schwabach, Germany) at 45 °C. The dried APE was collected, meticulously ground, and preserved at −40 °C until needed.

### 2.3. AG Solubility Determination

The solubility (30 ± 1 °C) of AG in different liquid vehicles, namely EtOH (volatile solvent-type liquid), NMP and Transcutol (non-volatile solvent-type liquids), and Labrasol ALF and Kolliphor EL (surfactant-type liquids), was assessed using a shake-flask methodology [43]. The solubility of AG in liquid vehicles containing solubilizers, specifically 1–3% *w*/*v* sodium dodecyl sulfate (SDS), was assessed. The solubility of AG at 37 ± 1 °C (dissolution test temperature) in various aqueous media, including deionized water, an acidic solution (0.1 N hydrochloric acid (HCl)), and solutions containing solubilizers, specifically 0.5% and 1% *w*/*v* SDS, as well as 1% and 2% *w*/*v* polysorbate P20 (P20), was also assessed using the same testing protocol. Briefly, a surplus of pAG powder was incorporated into 2 g of each liquid vehicle in a sealed tube. The resulting products were subjected to ultrasonic treatment for 60 min and maintained at the tested temperature (30 °C or 37 °C) in a shaking water bath for 24 h. The acquired supersaturated mixtures were processed using a 0.45 μm pre-filter membrane (Millipore, Burlington, MA, USA) and were diluted with methanol. The resulting solution was evaluated for its AG content using HPLC.

### 2.4. LS and Physical Mixture (PM) Powders of pAG and APEs

#### 2.4.1. Liquid Load Factor (L_f_) Determination

To ascertain the appropriate amounts of carrier (MCC) and coating (CSD) materials for the liquid vehicles, L_f_ was mathematically computed using a flowable liquid retention potential (Φ-value) model (Equation (1)) [44]. The Φ values of MCC (Φ_Ca_ value) and CSD (Φ_Co_ value) for the non-volatile liquid (NMP) were determined using the angle of slide evaluations. A fixed weight (0.3 g) of each powder excipient (MCC and CSD) was carefully blended with varying quantities of liquid vehicle, increasing in increments of 0.05 g, along with 0.5 g of volatile liquid (EtOH) to promote the uniform distribution of the liquid vehicle. The wet mass was allowed to rest at ambient temperature for 60 min, after which it was oven-dried at 50 °C for 30 min. Subsequent to each addition and drying, the angle of slide of the liquid-powder material mixture was assessed utilizing the inclined plane method with a tester constructed from stainless steel (Figure S1A, Supplementary Materials). The powder was then placed at one end of a surface-polished plate. The end was steadily elevated using a movable beam until the analyzed powder began falling off. The angle between the plate and the horizontal surface that induced the tested powder to slide was measured. The angle of slide for the NMP–powder mixture series was assessed through three repetitions.

A graph depicting the relationship between the weight ratio of the liquid vehicle to the powder and the related angle of slide was created. A trend line and corresponding linear equation were produced for each powder–liquid vehicle mixture. The coefficient of determination (R^2^) for the correlation was determined. The weight ratio of the liquid vehicle to the powder, which produced a sliding angle of 33°, was regarded as the flowable liquid retention capacity of the powder excipient, recorded as Φ_Ca_ and Φ_Co_ values for the examined liquid vehicles. The Φ_Ca_ and Φ_Co_ values were subsequently utilized to compute L_f_, employing an R value of 20, as per Equation (1).

(1) $$L_{f}=\Phi_{Ca}+\Phi_{Co}\cdot 1/R$$

(2) $$L_{f}=W/Q$$

(3) R=Q/q

where Φ_Ca_ and Φ_Co_ refer to the flowable liquid retention potentials of MCC and CSD, respectively. R signifies the weight ratio of the carrier (Q) to the coating material (q), and W represents the weight of the liquid medication [35,45,46].

#### 2.4.2. Fabrication of LS and PM Powders of pAG and APEs

LS powders of pAG (pAG–LS) and different APEs (APE–LSs) were formulated using an L_f_ value of 0.255 (0.255 g of NMP or NMP-based liquid medication per 1 g of MCC and 0.05 g of CSD). The details of these powdered formulations are listed in Table 1. To prepare the LS powder, the required quantities of pAG or APE were first dissolved in an appropriate amount of NMP. The pAG–NMP or APE–NMP mixture was ultrasonicated for 15 min to ensure complete dissolution of the solute. The resultant solution was uniformly blended with MCC for 10 min and then CSD for 10 min using a glass mortar and pestle. Blank LS powder (Blank–LS) was prepared by mixing the required amount of NMP with MCC for 10 min, followed by CSD for 10 min. For the PM powder, the required amount of pAG or APE was mixed with MCC (10 min) and then with CSD (10 min) using a glass mortar and pestle until a uniform mixture was obtained. The batch size for the preparation of both LS and PM powders was 100 g. The resulting LS and PM powders were collected without further sieving. All LS and PM powders were placed in separate sealed, light-proof containers and stored in a desiccator at refrigerated temperatures until use.

### 2.5. Characterization of LS and PM Powders of pAG and APEs

#### 2.5.1. Fourier Transform Infrared Spectroscopy (FTIR)

FTIR spectra of APEs from different sources were obtained and compared with those of pAG, PM, and LS powders. An ALPHA II FTIR spectrometer (Bruker Optics GmbH & Co. KG, Ettlingen, Germany) equipped with a platinum attenuated total reflection (ATR) accessory was used. The FTIR spectra of MCC, CSD, and NMP were also analyzed as controls. Each sample was directly placed as a thin layer on the ATR plate compartment for the measurements. Spectra were collected over the range of 4000–600 cm^−1^ with a resolution of 4 cm^−1^ and consisted of 32 consecutive scans at room temperature (24 ± 1 °C).

#### 2.5.2. Solid State Characterization

Powder X-ray diffraction (PXRD) was performed to elucidate the solid-state characteristics of APEs from various sources in comparison with pAG, as well as those of the PM and LS powders. PXRD patterns were acquired using an Empyrean diffractometer (Malvern Panalytical, Almelo, The Netherlands) equipped with Cu K-alpha radiation operating at 40 kV and 40 mA. The measurements were conducted across a range of 2θ values of 5 to 40° (0.02°/s).

#### 2.5.3. Morphological Study

The morphological and surface characteristics of the APEs of different origins, pAG powder, and their PM and LS powders were assessed using a Helios NanoLab G3 CX focused ion beam scanning electron microscope (Thermo Fisher Scientific, Hillsboro, OR, USA). Initially, each specimen was affixed to a metallic stub using double-sided carbon tape and gold sputter-coated under vacuum. Micrographs were captured using a backscattered electron detector at an acceleration voltage of 10 kV and magnification of 500× (for pAG, APEs, PM powders, and MCC-CSD mixture) and 1000× (for LS powders).

#### 2.5.4. Flow Properties

The flow characteristics of the PM and LS powders were assessed using two methodologies: Carr’s index and the angle of repose. To ascertain Carr’s index, the bulk and tapped densities of the powders were measured according to the procedure outlined in the USP (General Chapter 〈616〉 Bulk Density and Tapped Density) [48], with minor modifications. Specifically, 10 g of the test powder was precisely weighed and placed in a 25 mL cylinder. The initial volume was recorded to determine the sample bulk density. Subsequently, the powder-containing cylinder was tapped 250 times, and the resulting tapped volume was measured to determine the tapped density of the powder mixture. Carr’s index was calculated using Equation (4).

The fixed funnel method was used to determine the angle of repose of the samples. In this method, the test powder was gradually poured onto a flat surface using a glass funnel to form a conical pile of consistent height. The height (H, cm) and radius (R, cm) of the pile were measured, and the angle of repose was calculated using Equation (5).

*Carr’s index* (%) = ((*Tapped density* − *Bulk density*)/*Tapped density*) × 100 (4)

*Angle of repose* (°) = *Arctan* (*H*/*R*) (5)

#### 2.5.5. AG Content Determination

The quantities of AG in pAG and APEs, along with their PM and LS powders, equivalent to 3 mg of AG, were accurately measured and subsequently combined with 100 mL of absolute EtOH in a volumetric flask. The mixture was ultrasonicated for 0.5 h, followed by magnetic mixing for 20 h to ensure the complete extraction and dissolution of AG. The resultant combination was passed through a nylon syringe filter (0.45 μm pore size). AG content was evaluated using HPLC. Six replicates were performed for each sample analyzed.

#### 2.5.6. In Vitro Dissolution

The dissolution behaviors of AG from pAG, APEs, and their PMs, as well as the LS powders, were evaluated using a paddle apparatus (Erweka dissolution tester, Langen, Germany). The experiment employed 600 mL of dissolution medium maintained at 37 ± 0.5 °C. Two distinct dissolution media were used: 0.1 N HCl (pH 1.1 ± 0.1, *n* = 3) for the non-sink condition, and 0.5% SDS in 0.1 N HCl (pH 1.1 ± 0.1, *n* = 3) for the sink condition. Test samples, each containing an amount equivalent to 30 mg of AG, were precisely weighed and placed directly into the dissolution vessel assembly of the dissolution apparatus. The rotation speed was maintained at 50 ± 2 rpm. At specified time intervals (5, 10, 15, 30, 45, 60, 90, and 120 min) after adding the powder into the medium, 10 mL of the medium was withdrawn and replenished with fresh medium. The AG concentration was determined using HPLC.

Data analysis involved calculating and generating a graphical representation of the cumulative quantity of dissolved AG over time. To precisely determine the cumulative dissolved quantity of AG over time, adjustments for the sequential removal and replacement of 10 mL dissolution samples were implemented, and the mass of AG extracted at each sampling period was considered. The percentage of AG dissolved at 5 min (Q_5min_, %), which represents the cumulative amount of AG dissolved at a single point in time (5 min), was calculated. Dissolution profiles were also assessed through the dissolution efficiency (DE_120min_, %), as delineated by Khan and Rhodes [49], which was calculated from the area beneath the dissolution curves up to 120 min (Equation (6)). DE_120min_ was expressed as the percentage of the area of the rectangle corresponding to 100% dissolution within a 120 min time frame. Additionally, the similarity in the AG dissolution behaviors among the samples was evaluated using the similarity factor (*f*_2_), which was calculated using Equation (7).

(6) $$Dissolution\;efficiency,\;{DE}_{120min}=\frac{\int_{0}^{t}y\times dt}{y_{100}\times t}$$

(7) $$f_{2}=50\times log[\frac{1}{\sqrt{1+\left(\frac{1}{n}\right)\sum_{t=1}^{n}{\left(R_{t}-T_{t}\right)}^{2}}}\times 100]$$

where *y* represents the percentage of AG dissolved at a specific time (*t*), *n* signifies the number of sampling points, *R_t_* indicates the percentage of the reference (pAG, pAG–PM, or pAG–LS) that dissolved, and *T_t_* indicates the percentage of the test sample (APE, APE–PM, or APE–LS) that dissolved [43]. The mean value from all sampling time points of the six replicates was used for calculating *f*_2_. Notably, no time point exceeded 85% dissolution.

### 2.6. Stability Study

The physical and chemical stabilities of pAG and APEs, along with their PMs and LS powders, during storage were examined. Newly manufactured samples were enclosed in aluminum foil packaging bags and then kept in a refrigerator (5 ± 3 °C, refrigerated condition) and in KBF 240 Constant Climate Chambers (BINDER Inc., Bohemia, NY, USA) maintained at 30 ± 1 °C with 75 ± 5% relative humidity (RH) and at 40 ± 1 °C with 75 ± 5% RH, which refers to long-term and accelerated conditions for Climatic Zone IVb (hot and very humid environments) according to the ICH harmonized tripartite guideline for stability testing of new drug substances and products Q1A(R2) [50]. The specimens were maintained under these conditions for three months before analyzing their physical appearance, flowability, solid state (by PXRD), and remaining AG percentage. The dissolution behaviors of the aged LS powders were determined, and changes in the AG dissolution of the aged LS powders compared to that of the fresh samples were also examined using the *f*_2_ (Equation (7)) [43].

### 2.7. HPLC Methodology for AG Measurement

The AG and DDAG levels were measured using an HPLC system (LC-2050, Shimadzu, Kyoto, Japan) consisting of a photodiode array detector and a temperature control module, following the method of a previous report [51]. Separation was performed using an Agilent ZORBAX Eclipse Plus C18 (4.6 × 150 mm, 3.5 µm, Agilent Technologies, Inc., Santa Clara, CA, USA) controlled at 20 ± 0.5 °C. The detector was set at 254 nm. The mobile phase included 0.1% formic acid–acetonitrile (A) and a 0.1% formic acid–water solution (B). The gradient program measurements were as follows: 0–3 min, isocratic maintained at 30% A; 3–6 min, gradient to 40% A; 6–9 min, isocratic maintained at 40% A; 9–10 min, gradient to 80% A; 10–12 min, isocratic maintained at 80% A; 12–13 min, gradient to 30% A; 13–15 min, isocratic maintained at 30% A. The flow rate was adjusted to 1.4 mL·min^−1^, and the injection volume was 20 µL. Data analysis was performed using Shimadzu Class VP version 5.124 software (Shimadzu Corporation, Kyoto, Japan). As summarized in Table S1, the HPLC method demonstrated excellent linearity for both AG and DDAG (R^2^ = 0.9999). The method exhibited acceptable precision, with intra-day and inter-day %RSD values ranging from 1.38 to 3.97% and satisfactory accuracy, with recoveries ranging from 95.79% to 107.44%. The limits of detection (LODs) for AG and DDAG were 0.14 and 0.17 µg/mL, respectively, while the corresponding limits of quantification (LOQs) were 0.45 and 0.56 µg/mL, respectively. These validation results complied with the AOAC International guidelines for analytical method validation [52].

### 2.8. Statistical Analysis

Quantitative results are presented as the mean ± standard deviation (SD). Microsoft Excel was used to compute the means and SDs and to plot graphs for each experiment. One-way analysis of variance (ANOVA) followed by Tukey’s test was performed using SPSS software for MS Windows (version 19; SPSS (Thailand) Co., Ltd., Bangkok, Thailand) to analyze the significance of the differences in the dissolution parameters (*n* = 6) and the percentage of AG remaining in the samples after storage (*n* = 6). In all statistical analyses, a *p*-value of less than 0.05 was considered to indicate a significant difference between the groups.

## 3. Results and Discussions

### 3.1. APEs: AG Content and Morphology

In this study, APEs were successfully prepared using Soxhlet extraction of *A. paniculata* leaves with absolute EtOH. The APE yields were 8.23%, 5.03%, and 6.85% for APE–S1, APE–S2, and APE–S3, respectively. AG was the predominant diterpene lactone, and DDAG was the second most abundant diterpene lactone in all three prepared APEs. The AG contents were 24.13 ± 1.11%, 17.07 ± 1.37%, and 14.88 ± 1.23% for APE–S1, APE–S2, and APE–S3, respectively. The DDAG contents were 4.07 ± 0.09%, 5.76 ± 0.05, and 13.84 ± 1.15% for APE–S1, APE–S2, and APE–S3, respectively. These results are related to the diterpene lactone content of the herbal materials. The AG levels in the Andrographis material used in this study align with previous findings that identified diterpene lactones in *A. paniculata* leaves collected from different regions in Thailand [15].

**Figure 1:**
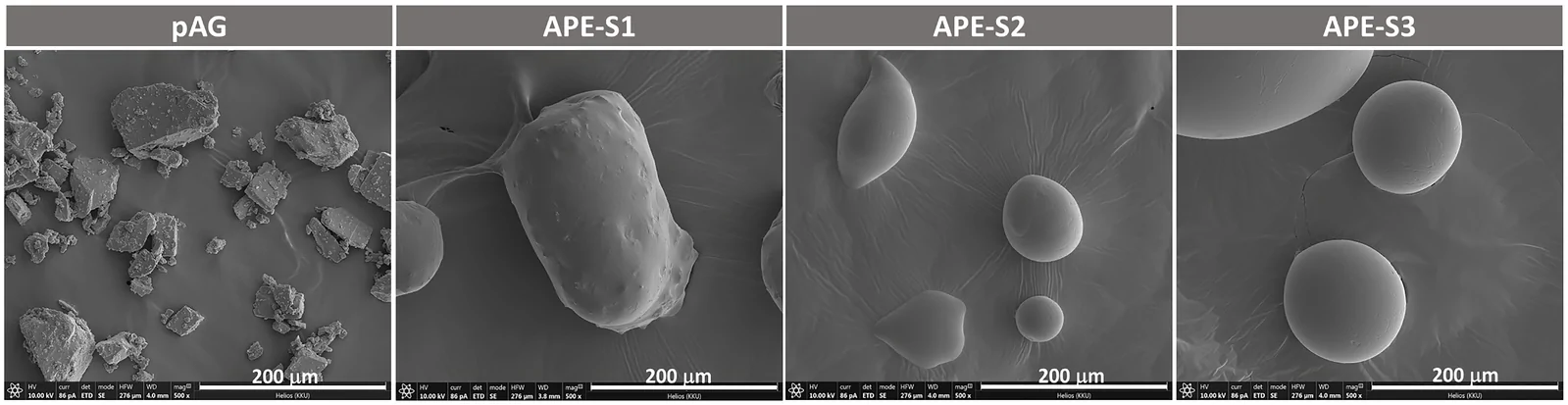
SEM photographs (500×) of pAG and APEs obtained from various sources. Abbreviations: APEs—*Andrographis paniculata* extracts; APE–S1—*Andrographis paniculata* extract derived from source 1; APE–S2—*Andrographis paniculata* extract derived from source 2; APE–S3—*Andrographis paniculata* extract derived from source 3; pAG—pure andrographolide powder.

All the obtained APEs were dry, hygroscopic solids characterized by a dark green color, whereas the pAG powder was a fine white powder. The SEM images are shown in Figure 1. The crystalline characteristics of the pAG particles were evident and consistent with those of previous studies [13,53]. The APEs appeared as smooth-surface droplet-shaped solids, except for APE-S1, which displayed a rough surface. This coarse surface texture appeared to incorporate solid particles. It is important to highlight that there is limited documentation on APE morphology.

### 3.2. AG Solubility

**Figure 2:**
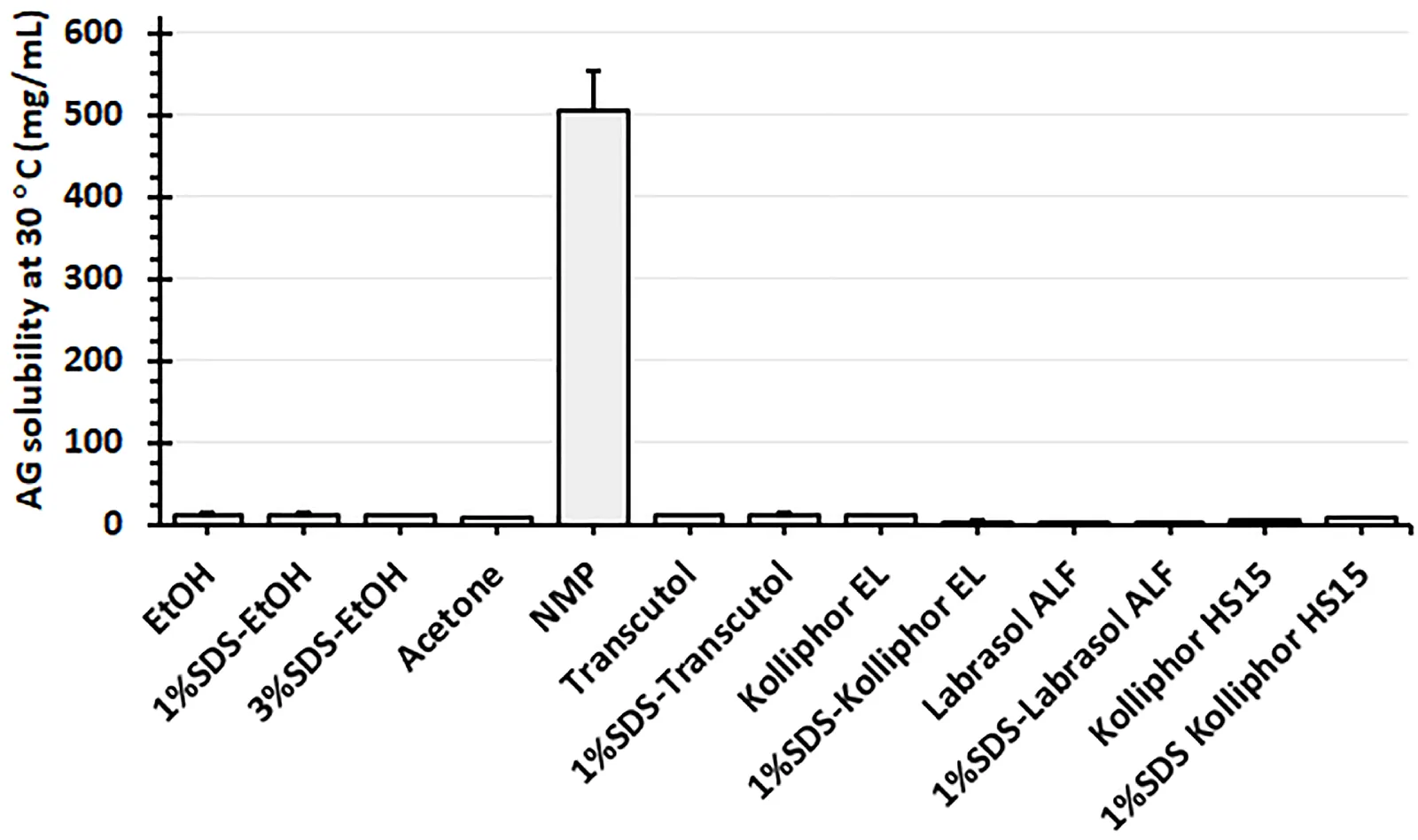
Solubility values of pAG in various tested liquid solvents at 30 °C with and without SDS. Each value represents the mean ± SD of *n* = 3 samples. Abbreviations: EtOH— ethyl alcohol (99.8%); NMP—N-methyl-2-pyrrolidone; pAG—pure andrographolide powder; SDS—sodium dodecyl sulfate.

The solubility of AG in numerous liquids was investigated using pAG via the shake flask method. This methodology remains a conventional and widely applicable technique for determining the solubility [54] of pharmaceutical and phytopharmaceutical compounds, including AG [22,28,55,56]. The results indicated that pAG exhibited limited solubility in most of the investigated solvents (Figure 2). The highest pAG solubility was observed in NMP (507 ± 47 mg/mL). Considering the reported density of NMP of 1.0217 g/mL at 30 °C [57], the average AG solubility in NMP in terms of weight percentage would be ~33.2% *w*/*w*. In other solvent systems, the solubility of AG was less than 13 mg/mL. The incorporation of SDS, an anionic surfactant, into the liquid vehicle, either a nonvolatile (Transcutol, Labrasol ALF, and Kolliphor EL) or volatile (EtOH) solvent, did not improve the solubility of pAG in the liquid solvents. The superior solubility of pAG in NMP compared to that in other solvents is consistent with the results of previous studies [28]. Liu et al. [28] found that NMP was capable of achieving a maximum solubility value of up to 26% *w*/*w* at a temperature of 25 °C. The limited solubility of AG in the other tested liquids was similar to previously reported values. Prior studies have shown that AG exhibits poor solubility in oils, including coconut, corn, olive, and soybean oils, oleic acid, and α-tocopherol, with a solubility value of ≤2.5 mg/mL (37 °C) [22,56]. In nonionic surfactants such as Span 80, Cremophore EL, Tween 80, and Tween 20, the AG solubility was also limited to ≤3.1 mg/mL (37 °C) [55]. Nonpolar solvents, including Transcutol HP and polyethylene glycol 400, and semipolar solvents, such as propylene glycol and glycerin, also provide a low solubility value of less than 54 mg/mL (37 °C) [22].

The solubility of AG in liquid vehicles can be attributed to their solute–solvent interactions. Liquids that interact with solutes through hydrophobic forces, hydrogen bonding, and van der Waals forces are generally effective solvents. This could be attributed to the powerful solvency capacity of NMP for AG. The solubility of the AG compound in NMP was sufficiently high; hence, this solvent was considered appropriate for the pAG–LS and APE–LS powders in this study. The liquid with the greatest solvency capacity for the loaded active ingredients is generally used as the liquid vehicle in LS systems because of its ability to induce complete molecular drug dispersion yielding a fraction of molecularly dispersed drug parameter value of 1 [30]. NMP has been successfully used as a liquid vehicle for numerous LS systems, including those with AG compounds [28,29,58,59].

NMP is regarded as a powerful solvent and solubilizer for parenteral, oral, and topical pharmaceutical preparations [60,61]. Several NMP-based drug delivery systems with enhanced dissolution and oral absorption of poorly water-soluble active ingredients, including liquisolid and self-nanoemulsifying drug delivery systems, have been developed [28,29,58,59,62,63]. Patented oral formulations that include NMP have also been reported, which enhanced solubility and/or dissolution, leading to improved bioavailability [64,65]. NMP is included in numerous pharmacopeias, which have established rigorous quality standards and monographs for pharmaceutical-grade NMP (methylpyrrolidone) [48]. The United States Food and Drug Administration has categorized NMP as a class 2 solvent and established a daily exposure limit of 5.30 mg/day [60]. Nevertheless, NMP exhibits relatively low acute oral toxicity in experimental animals, with reported oral LD_50_ values exceeding 3 g/kg body weight [29,66]. Nonetheless, the suitability of NMP as an oral excipient in oral pharmaceutical formulations, particularly in the context of chronic or repeated exposure, presents regulatory concerns. Repeated intake of high doses (approximately 250–500 mg/kg body weight/day) has been shown to trigger undesirable reproductive effects in experimental animals [67]. Sub-chronic exposure of male rats conducted by Sitarek and Stetkiewicz [68] revealed that NMP, at the maximum dosage examined (1000 mg/kg body weight/day), induced significant male infertility and considerable pathomorphological alterations in the seminiferous epithelium in the testicular seminal tubules, disrupting normal spermatogenesis. Doses at or below 300 mg/kg body weight/day did not significantly affect male mating success, fertility metrics, or the structural integrity of spermatogenesis; however, only the lowest dose of 100 mg/kg body weight/day had no effect on the prenatal development of progeny.

### 3.3. pAG and APE Powder Characteristics

#### 3.3.1. L_f_ for LS Powder

To fabricate the LS powder, liquid medications carrying the active components, pAG and APEs, were deposited onto the structures of the carrier and coating materials. Given that powdered substances may only contain limited quantities of liquid components, the L_f_ of the 20:1 MCC–CSD system was evaluated in this study. L_f_ denotes the mass ratio of the liquid medication to the carrier material (Equation (2)), which must exhibit adequate flow properties and compressibility. L_f_ was determined based on the greatest amount of liquid vehicle that could be maintained per unit mass of the powder excipient while maintaining an adequate flowability [44,46]. To evaluate the flow properties of powders that incorporate nonvolatile liquid vehicles, a specific parameter, the angle of slide, is primarily used. According to the mathematical models proposed by Spireas et al. [44], the maximum amount of a non-volatile liquid vehicle that a unit mass of carrier and coating material can retain while maintaining adequate flowability, denoted as Φ_Ca_ and Φ_Co_, respectively, is determined at an angle of slide of 33°. This threshold is regarded as the upper limit at which liquid-loaded powder admixtures transition from acceptable sliding behavior to cohesive resistance and serves as a critical preformulation parameter to ensure satisfactory powder flow. Based on the regression formula correlating the average value of the angle of slide (y-axis) and the corresponding weight ratio of NMP to MCC (X-axis) (y = 16.203x + 29.608 (R^2^ = 0.936)), the Φ_Ca_ value for NMP was determined to be 0.209. Considering the regression formula correlating the average value of the angle of slide (y-axis) and the corresponding weight ratio of NMP to CSD (X-axis) (y = 4.780x + 28.627 (R^2^ = 0.866)), the Φ_Co_ value was found to be 0.915 (Figure S1B, Supplementary Materials). This resulted in an L_f_ value of MCC–CSD with an R-value of 20 of 0.255. The reported L_f_ values of MCC–CSD (R = 20) are generally modest, ranging from 0.176 to 0.315, depending on the liquid solvent used [39,41,69].

In the present study, NMP was employed primarily to improve the solubilization of AG in the liquisolid formulations. Despite strict toxicological thresholds, NMP is actively utilized as an excipient in several regulatory-approved parenteral (injectable) formulations for humans and animals [60]. An in situ implant marketed for subcutaneous injection of leuprolide acetate (Sanofi Aventis, Quebec, Laval, Canada) was developed with NMP at a concentration of 55–66% as a co-solvent [62]. According to the Thai Herbal Pharmacopoeia [70], the recommended daily dose of powdered *A. paniculata* is 2–3 g/day, corresponding to an AG intake of approximately 20–30 mg/day, based on the pharmacopeial requirement of not less than 1% *w*/*w* AG. Based on the formulation composition, administration of a dose equivalent to 30 mg of AG would result in an NMP exposure of approximately 271 mg for pAG-LS and 187–303 mg for the APE-LS formulations, depending on the AG content of the APEs. Although the calculated NMP exposure exceeded the current exposure limit established for residual solvents, it remained substantially lower than the dose level associated with reproductive toxicity following repeated administration in experimental animals [68]. Importantly, this study demonstrates the utility of NMP as a model solvent in the LS systems for elucidating the solvation requirements of AG and reducing the variability in the dissolution of AG from different APE sources.

The concentration of pAG in the liquid medications was maintained at 10%, while that of APEs was maintained at 40% *w*/*w*, which corresponds to AG concentrations ranging between 6.0% and 9.97% *w*/*w*, depending on the AG level contained in the pAG and APEs. For all LS powder formulations, the AG concentrations in the liquid medication (pAG-NMP or APE-NMP) were clearly lower than the solubility value of AG in NMP (estimated as 33.2% *w*/*w*) by 3.3–5.6 fold (Table 1). These ratios led to an estimated fraction of molecularly dispersed AG in LS powder equal to 1, indicating that AG can be fully dissolved in NMP [47,71]. Previous reports have shown that LS systems with FM values of 1.0 effectively enhance dissolution [29,47,72,73].

Previous reports suggest that MCC and CSD are the most frequently employed excipients in LS formulations owing to their long-standing use in solid dosage forms and their stability. The large particle size of MCC enhances the favorable flow characteristics of the powders. A small amount of nanoscale CSD coats and adsorbs excess liquid on the MCC surface, contributing to system dryness. The moderate specific surface area of approximately 1.18 m^2^/g for MCC is improved by the significantly higher specific surface area of approximately 200 m^2^/g for CSD [16,35]. This ultimately enhances a particular aspect of the system, thereby increasing its liquid load capacity. An MCC–CSD mixture with an R-value of 20 is commonly used for LS formulations [74,75,76]. Although CSD can function as a glidant, a large amount can reduce powder system flowability due to its very fine particles with a bulky character [77,78].

#### 3.3.2. Appearance, Flowability, and AG Content

**Figure 3:**
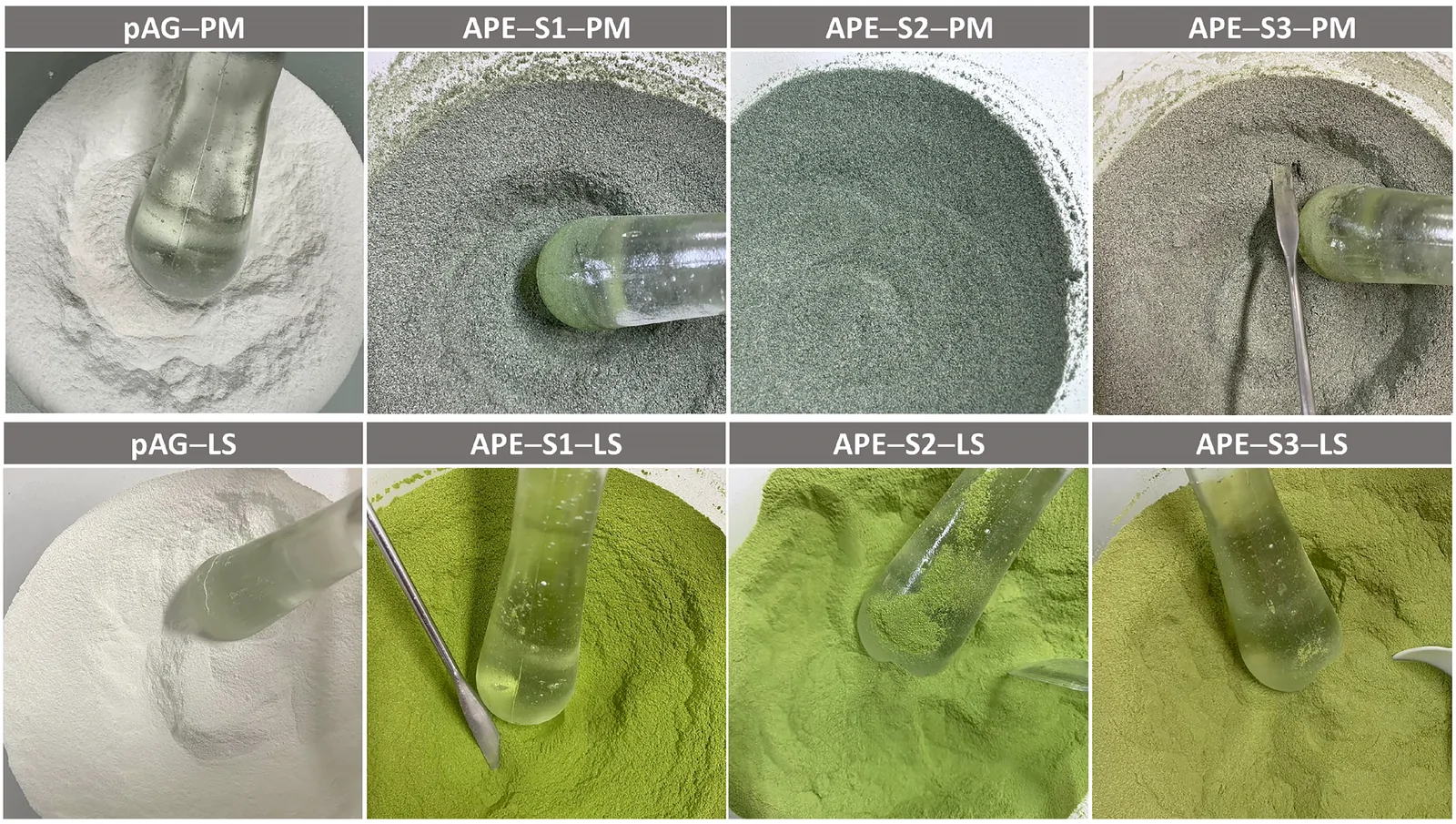
Physical appearances of PM and LS powders of pAG and APEs. Abbreviations: APEs—*Andrographis paniculata* extracts; APE–S1—*Andrographis paniculata* extract derived from source 1; APE–S2—*Andrographis paniculata* extract derived from source 2; APE–S3—*Andrographis paniculata* extract derived from source 3; LS—liquisolid; pAG—pure andrographolide powder; PM—physical mixture.

Based on the established L_f_ values, pAG–LS and APE–LS powders were developed. In the preparation of the LS powder, NMP was used to dissolve either pAG or APE prior to their deposition onto solid particles. These particles, specifically MCC and CSD, functioned as the carrier and coating materials, respectively (Table 1). For control purposes, pAG and APE powders were used in the form of PMs. As illustrated in Figure 3, a uniform APE–PM powder was achieved by manually pulverizing the APEs and solid particles (MCC and CSD). Distinct APEs exhibit different green tones. All APE–LS powders exhibited a dry, homogeneous consistency and green color. This indicates the effective and uniform incorporation of APEs onto the solid particles. The different APEs exhibited different shades of green. A dry, homogeneous white powder was obtained for both pAG–PM and pAG–LS. It is important to highlight that the LS and PM powders produced in this study were not sieved after their preparation. Typically, the sieving process is unnecessary for the manufacture of LS powders as a dry and free-flowing powder is achieved when an appropriate L_f_ value is used. However, as the particle size of the powder mixture may affect the powder characteristics and dissolution, the particle size range of the final product should be determined.

The powder flow of all pAG and APE powders was examined using Carr’s index and the angle of repose, and the results are listed in Table 2. The Carr’s index values of the PM powders ranged from 9.8% to 16.4%, indicating fair-to-excellent flowability. The Carr’s index for the LS powders (10.3–14.2%) indicates that these powders exhibited good to excellent flowability and compressibility [48]. The angle of repose values for all investigated powders, reaching up to 34.4°, were deemed to exhibit good-to-excellent flow characteristics. LS powders are primarily designed for the production of compacts or capsules, where flowability and compressibility are essential quality attributes. Powder flowability is crucial in numerous pharmaceutical manufacturing processes, including the production of granules, tablets, and capsule fillings [79]. Consequently, optimal flowability of APE powders is essential to ensure a uniform and consistent transfer of materials from bulk storage containers to the feeder throughout production.

Carr’s index serves as a metric for assessing the frictional and cohesive characteristics of powders under static settings. The angle of repose quantifies the dynamic friction properties of powdered materials. Cohesive particles exhibit increased values for both Carr’s index and the angle of repose [80,81]. The flowability data suggested that the prepared pAG and APE powders were dry and noncohesive. A dry and free-flowing LS powder, despite containing a liquid vehicle, can generally be achieved when the L_f_ of the liquid medication, specifically a solution of pAG or APE in NMP, does not exceed the L_f_ of the carrier–coating system. However, in terms of the angle of repose, the less favorable flowability of pAG-LS compared to that of the corresponding PM may be attributed to the greater amount of NMP liquid in pAG-LS than in APE-LS powders (Table 1). This larger amount of NMP may have affected the dynamic friction properties of the powder.

The AG contents of the pAG and APE powders were analyzed, and the results are presented in Table 2. Notably, the theoretical AG levels in the APE-LS and APE-PM powders varied between 12.07 and 19.52 mg/g and between 59.60 and 96.40 mg/g, respectively, depending on the AG content of the respective APEs. The measured values of AG content in the resulting LS and PM powders ranged from 95.5 to 97.3% and 95.4 to 101.8%, respectively, of the corresponding theoretical loadings. The small SD values of the measured AG percentages indicated the consistency of the AG content in both pAG and APE powders. The application of pAG or APEs onto carrier-coated particles through direct trituration (PM powder) or dissolution with NMP (LS powder) resulted in a homogeneous dispersion of pAG or APEs on the carrier–coating surface. This process facilitated a uniform distribution of AG molecules across the batches. The homogeneous distribution of phytopharmaceuticals in LS formulations has been extensively studied [28,34,35,36].

#### 3.3.3. Morphological and Solid State

**Figure 4:**
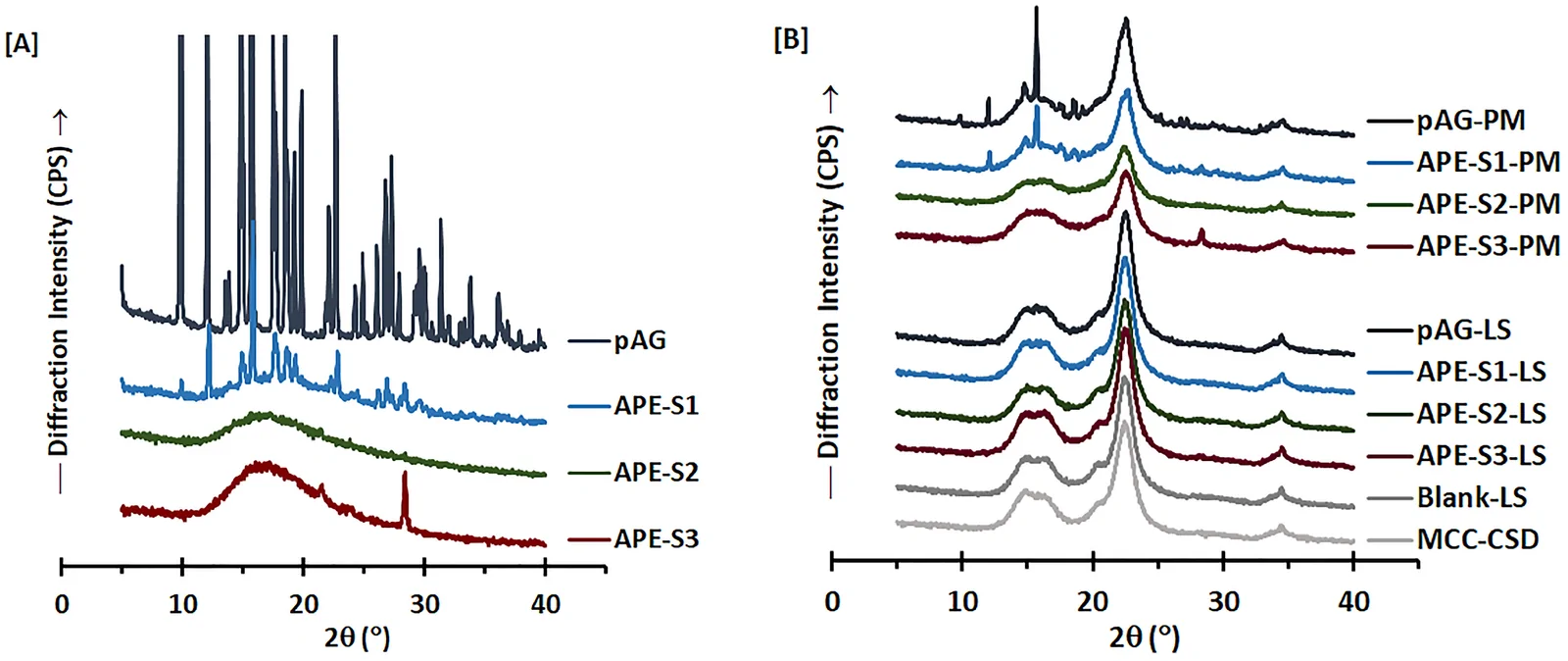
PXRD patterns of pAG and different APEs (**A**), their PM and LS powders, Blank–LS, and MCC–CSD (20:1 MCC:CSD mixture) (**B**). The arrow indicates the diffraction intensity direction. Abbreviations: APEs—*Andrographis paniculata* extracts; APE–S1—*Andrographis paniculata* extract derived from source 1; APE–S2—*Andrographis paniculata* extract derived from source 2; APE–S3—*Andrographis paniculata* extract derived from source 3; CSD—colloidal silicon dioxide; LS—liquisolid; MCC—microcrystalline cellulose; pAG—pure andrographolide powder; PM—physical mixture.

Figure 4A shows the PXRD patterns of the APEs in comparison with that of pAG. pAG exhibited pronounced diffraction peaks at 2θ values ranging from 9.8° to 39.6°. This finding is consistent with previous reports [21,28,82,83]. The differences in the PXRD patterns of the APEs obtained from various sources were clearly observed. APE–S1 demonstrated significant crystal diffraction peaks at the positions of 9.9°, 12.2°, 14.9°, 15.8°, 17.7–17.8°, 18.6°, 19.4°, 22.8°, and 26.9° 2θ. This indicates the inherent crystallization properties of AG compound. The PXRD pattern of APE–S1 aligned with the SEM images, indicating the presence of crystalline particles in the extract. APE–S1 exhibited an additional peak at 28.4° 2θ, which was different from that of pAG. The PXRD pattern of APE–S2 exhibited a somewhat broad profile, featuring a minor peak alongside significant peaks at approximately 15.7°, 21.5°, and 28.4° 2θ. The PXRD pattern of APE–S3 revealed minor peaks related to AG characteristics, alongside two significant diffraction peaks at 21.5° and 28.4° 2θ. The existence of an alternate crystalline compound in these APEs is indicated by the occurrence of peaks, such as at approximately 21.5° and 28.4° 2θ, which do not correspond with those of the AG compound.

The PXRD patterns clearly indicate differences in the solid states of different APEs. The PXRD data suggest that the compounds, particularly AG, present in APE–S1 may exist in a crystalline form. This may be associated with the elevated AG content in APE–S1 and the extraction protocol used. The AG content in APE–S1 (24.13 ± 1.11%) was approximately 1.4 and 1.7 times higher than that in APE–S2 (17.07 ± 1.37%) and APE–S3 (14.88 ± 1.23%), respectively. The low solvent ratio of absolute EtOH to *A. paniculata* dried leaf powder (6:1) used in Soxhlet extraction might result in a supersaturation state of AG once the APE–S1 extract was cooled to room temperature. Previous studies have indicated that the solubility of AG in EtOH increases with temperature [84]. It can be inferred that AG exhibited greater solubility at an extraction temperature of 60 °C compared to room temperature. This may initiate the nucleation of AG crystals created after a temperature reduction, subsequently leading to crystal growth. Moreover, AG crystals may form upon concentration via solvent evaporation. It is known that numerous chemicals found in plant extracts are crystalline. The crystallization of substances may occur during the concentration of an extract using heat or refrigeration [85]. Currently, there is a shortage of studies examining the solid-state of AG from various APE sources using PXRD.

Figure 4B demonstrates the solid states of the pAG and APE powders compared with those of MCC–CSD and Blank–LS. The diffractogram of MCC–CSD exhibited a mixed pattern indicative of the semicrystalline structure of MCC, characterized by a prominent sharp peak at a 2θ diffraction angle of 22.4°, a broad peak centered at 16°, and a minor peak at 34.5° of MCC, along with the amorphous halo pattern of CSD [35,36]. The PMs exhibited the distinct patterns of pAG or APEs, along with those of MCC–CSD. This implies that the pulverization of pAG or APEs with MCC–CSD did not influence the solid state of the pAG or APEs. Distinctive peaks of the AG compound in pAG–LS or any other crystalline compound from the APEs in APE–LS, regardless of the APE source, were absent in the spectra. The absence of characteristic AG peaks in the PXRD patterns of the pAG-LS and APE-S1-LS powders may result from the molecular dissolution or non-crystalline state of AG in the LS systems. The potential transformation of AG to a non-crystalline state could be attributable to AG having a greater saturated solubility (S_AG_) than its concentration (C_AG_) within the liquid medication, pAG-NMP or APE-NMP (Table 1). The conversion of crystalline drugs and phytopharmaceuticals to a non-crystalline form via LS technology has also been observed in previous reports [16,28,29,31,36,86,87]. Nevertheless, it should be noted that the absence of diffraction peak characteristics in the PXRD patterns may also result from the low concentration of the crystallite below the PXRD detection limit and/or peak masking by the excipients. Further evaluations are needed to investigate and confirm the molecular dispersion state of AG molecules, including their thermal behavior via differential scanning calorimetry, as well as a quantitative assessment of the PXRD detection limit. Additionally, separate studies on the solid-state transformation induced by LS technology using pure compounds should also be extended to the other diterpene lactone marker, DDAG.

**Figure 5:**
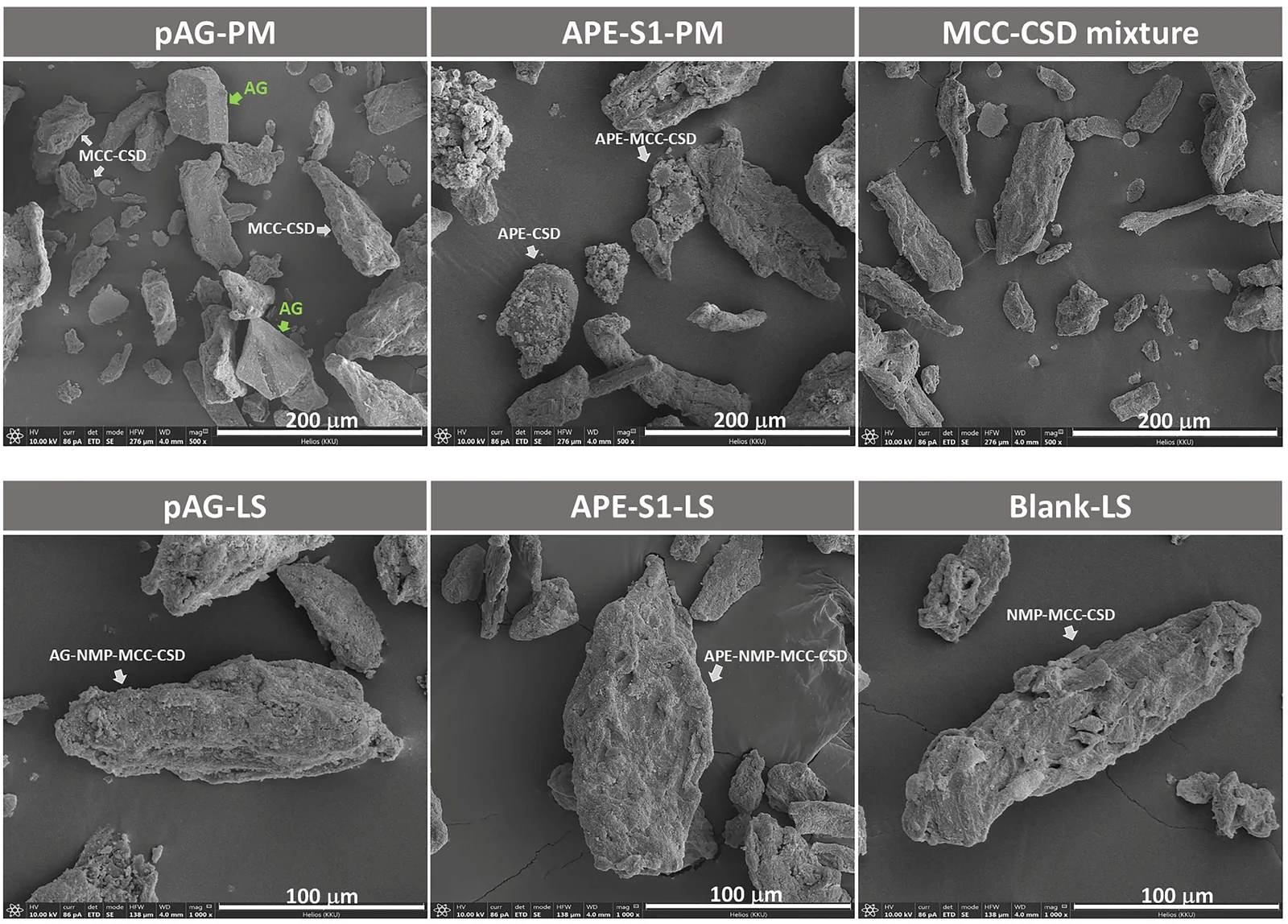
SEM photographs of PM (500×) and LS powders (1000×) of pAG and APE–S1, along with the MCC–CSD mixture (20:1 MCC:CSD ratio) (500×) and Blank–LS (1000×). Abbreviations: APE—*Andrographis paniculata* extract; APE–S1—*Andrographis paniculata* extract derived from source 1; CSD—colloidal silicon dioxide; LS—liquisolid; MCC—microcrystalline cellulose; NMP—N-methyl-2-pyrrolidone; pAG—pure andrographolide powder; PM—physical mixture.

The morphologies of the AG and APE powders were studied using SEM and compared with those of the MCC–CSD particles and Blank–LS (Figure 5). The MCC–CSD mixture displayed micron-sized cellulose fragments with a highly textured surface of MCC coated with nanosized CSD particles. The pAG–PM comprised pAG particles characterized as irregularly shaped crystalline particles with defined edges (Figure 1), along with MCC particles. Their surfaces were covered with CSD particles. Similarly, APE–PM demonstrated the characteristics of APE particles combined with MCC and coated with CSD. Furthermore, the significant surface thickening of the MCC particles may be associated with the deposition of ground APE onto the MCC–SD mixture. In the case of LS powders, the absence of pAG particles, APE particles, or APE fragments on the MCC was observed. The modest thickening and smoothing of the MCC particle surface were linked to the deposition of the pAG–NMP or APE–NMP solution on the MCC. Blank–LS, in which only the NMP vehicle was applied to the MCC–CSD particles, displayed slightly smoother surfaces than the MCC–CSD powder. Notably, no discernible differences were evident between the LS powders of pAG and APE from various sources. Additionally, all APE–LS powders displayed analogous morphological appearances, regardless of the APE source.

#### 3.3.4. Compatibility

**Figure 6:**
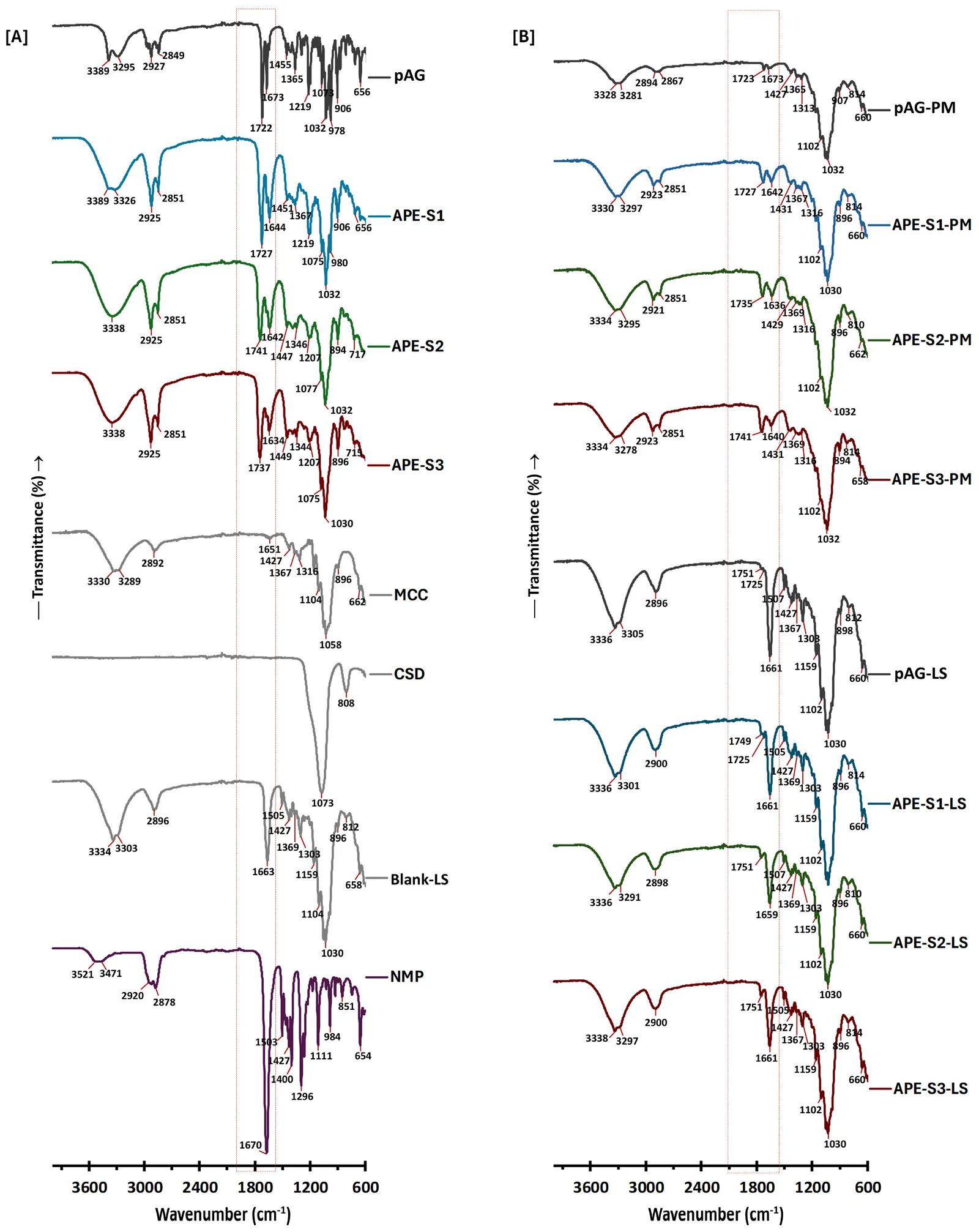
FTIR patterns of pAG, APEs, MCC, CSD, Blank–LS, and NMP solvent (**A**), and pAG–PM, APE–PM, pAG–LS, and APE–LS (**B**). Abbreviations: APEs—*Andrographis paniculata* extracts; APE–S1—*Andrographis paniculata* extract derived from source 1; APE–S2—*Andrographis paniculata* extract derived from source 2; APE–S3—*Andrographis paniculata* extract derived from source 3; CSD—colloidal silicon dioxide; LS—liquisolid; MCC—microcrystalline cellulose; NMP—N-methyl-2-pyrrolidone; pAG—pure andrographolide powder; PM—physical mixture.

ATR-FTIR analysis was conducted to examine the compatibility between AG or the constituents of the APEs and the LS components, with the results illustrated in Figure 6. pAG exhibited a broad band with peaks at 3389 cm^−1^ and 3295 cm^−1^, which correspond to O–H stretching vibrations, along with peaks in the range of 2850–2960 cm^−1^, which are indicative of –C–H stretching of methyl and methylene (aliphatic) groups. The strong peaks at 1722 cm^−1^ and 1673 cm^−1^ were assigned to the C=O stretching in the α,β-unsaturated lactone ring and C=C stretching (conjugated alkene within the lactone system), respectively. Peaks within the range of 1450–1370 cm^−1^ corresponded to the bending vibrations of the CH_2_ and CH_3_ groups. Peaks between 1250 cm^−1^ and 1050 cm^−1^ were attributed to the stretching of C–O–C bonds, indicative of ether or lactone structures. The 950–900 cm^−1^ region was associated with the out-of-plane bending of =C–H, which is characteristic of alkenes [13,83,88,89]. All APEs exhibited FTIR patterns analogous to those of pAG, with slight variations in the peak positions and intensities. The broad band at 3050–3600 cm^−1^ indicates the –O–H stretch of alcohols and phenols. Peaks around 2800–3000 cm^−1^ (C–H stretching) and approximately 1450–1380 cm^−1^ (C–H bending) indicate the presence of alkyl chains (CH_2_ and CH_3_). A pronounced absorption in the range of 1700–1750 cm^−1^, corresponding to the –C=O stretching vibration, suggests the presence of an α,β-unsaturated lactone ring in the structure.

The presence of multiple peaks in the 1300–1000 cm^−1^ range, indicative of C–O bonds, substantiates the identification of esters. The presence of aromatic compounds or alkenes was indicated by the C=C stretching vibrations and out-of-plane C–H bending modes observed below 900 cm^−1^ [88,90,91,92] (Figure 6A).

The ATR-FTIR spectrum of MCC displayed distinctive O–H stretching at 3330 and 3289 cm^−1^, C–H stretching at 2892 cm^−1^, CH_2_ bending at 1427 cm^−1^, C–H bending at 1367 cm^−1^, C–OH stretching at 1058 cm^−1^, and β-glycosidic bond C–H deformation at 896 cm^−1^. A minor signal at 1651 cm^−1^ corresponds to the H–O–H bending of the adsorbed water molecules in the MCC (Figure 6A). The ATR-FTIR spectra of the CSD displayed distinctive peaks for the asymmetric and symmetric stretching of Si–O–Si at 1073 cm^−1^ and 808 cm^−1^, respectively (Figure 6A) [35]. All PM powders exhibited a superposition of the characteristic ATR-FTIR bands of MCC, CSD, and pAG (for pAG–PM) or APEs (for APE–PM), without any notable peak shifts. These findings indicate a lack of interaction among the components of the PM produced by trituration (Figure 6B).

As depicted in Figure 6A, the ATR-FTIR spectra of NMP exhibited absorption between 3000 and 2800 cm^−1^, characteristic of C–H stretching in aliphatic compounds and N–CH_3_ groups. The sharp band at 1670 cm^−1^ corresponds to the C=O stretching vibration of the lactam carbonyl group [93]. Blank–LS exhibited the characteristic bands of MCC and CSD, in conjunction with NMP bands. Notable shifts of the C=O stretching band of NMP to lower wavenumbers, from 1670 cm^−1^ to 1663 cm^−1^ (wavenumber difference = 7 cm^−1^), were clearly observed. Shifts of O–H stretching to 3334 cm^−1^ and 3303 cm^−1^, along with C–OH stretching to 1030 cm^−1^ of MCC were also observed in the Blank–LS spectrum. This suggests the presence of intermolecular interactions, potentially involving hydrogen bonding between the carbonyl group of NMP and the hydroxyl groups on the cellulose chains of MCC.

pAG-LS and APE-LSs also exhibited persistent shifts in the C=O stretching band of NMP, as well as in the O–H and C–OH stretching bands of MCC. For pAG-LS, a shift in the absorption of the band associated with the C=O stretching within the α, β-unsaturated lactone ring of the AG molecules from 1722 to 1751 cm^−1^ (wavenumber difference = 29 cm^−1^), with a minor peak persisting at 1725 cm^−1^, was observed. In addition, a shift of the C=O stretching band of NMP to lower wavenumbers, from 1670 cm^−1^ to 1661 cm^−1^ (wavenumber difference = ~10 cm^−1^), was observed. To clarify whether these shifts were related to the association between pAG and NMP, the FTIR spectra of 10% *w*/*w* pAG in NMP solution were assessed (Supplementary Materials, Figure S2). A similar pattern to that observed in the pAG-LS spectrum was revealed, including a shift in the C=O stretching absorption band of the AG molecules from 1722 to 1752 cm^−1^ (wavenumber difference = 30 cm^−1^) with a shift of the C=O stretching band of NMP to lower wavenumbers, from 1670 cm^−1^ to 1665 cm^−1^ (wavenumber difference = 5 cm^−1^). This suggests the existence of intermolecular interactions between AG and NMP. Shifts in these C=O stretching bands were also observed for the APE-LS samples. For APE-S1-LS, the C=O stretching absorption band of APE-S1 shifted towards higher wavenumber, from 1727 to 1749 cm^−1^, a wavenumber difference of 22 cm^−1^, with a shift of the C=O stretching band of NMP to lower wavenumbers, from 1670 cm^−1^ to 1661 cm^−1^ (wavenumber difference = ~10 cm^−1^). For APE-S2-LS, a shift in the C=O stretching absorption band of APE-S2 from 1737 to 1751 cm^−1^ (wavenumber difference = 14 cm^−1^) was observed, along with a shift of the C=O stretching band of NMP to lower wavenumbers, from 1670 cm^−1^ to 1659 cm^−1^ (wavenumber difference = ~12 cm^−1^). In the case of APE-S3-LS, there was a shift in the C=O stretching absorption band of APE-S3 from 1741 to 1751 cm^−1^ (wavenumber difference = 10 cm^−1^) with a shift of the C=O stretching band of NMP to lower wavenumbers, from 1670 cm^−1^ to 1661 cm^−1^ (wavenumber difference = ~10 cm^−1^). Notably, the extent of the wavenumber shift of the C=O stretching band in the α,β-unsaturated lactone ring of the three distinct APEs appears to correlate with the AG concentration in the APE (Section 3.1), with APE-S1-LS (24.13% AG content) having the greatest wavenumber shift to a higher wavenumber and APE-S3-LS (14.88% AG content) having the smallest wavenumber shift. The unique wavenumber shifts observed in APE-LSs align closely with those of the pAG-NMP solution and pAG-LS, indicating the presence of intermolecular interactions between AG in APEs and NMP. Nevertheless, APEs contain other diterpene lactones, specifically DDAG, as well as numerous other chemical constituents. Such interactions can also occur between diterpenes and compounds from other chemical groups, such as phenolics. To confirm the molecular interactions observed via FTIR shifts, difference spectroscopy was applied.

#### 3.3.5. Dissolution Behaviors

**Figure 7:**
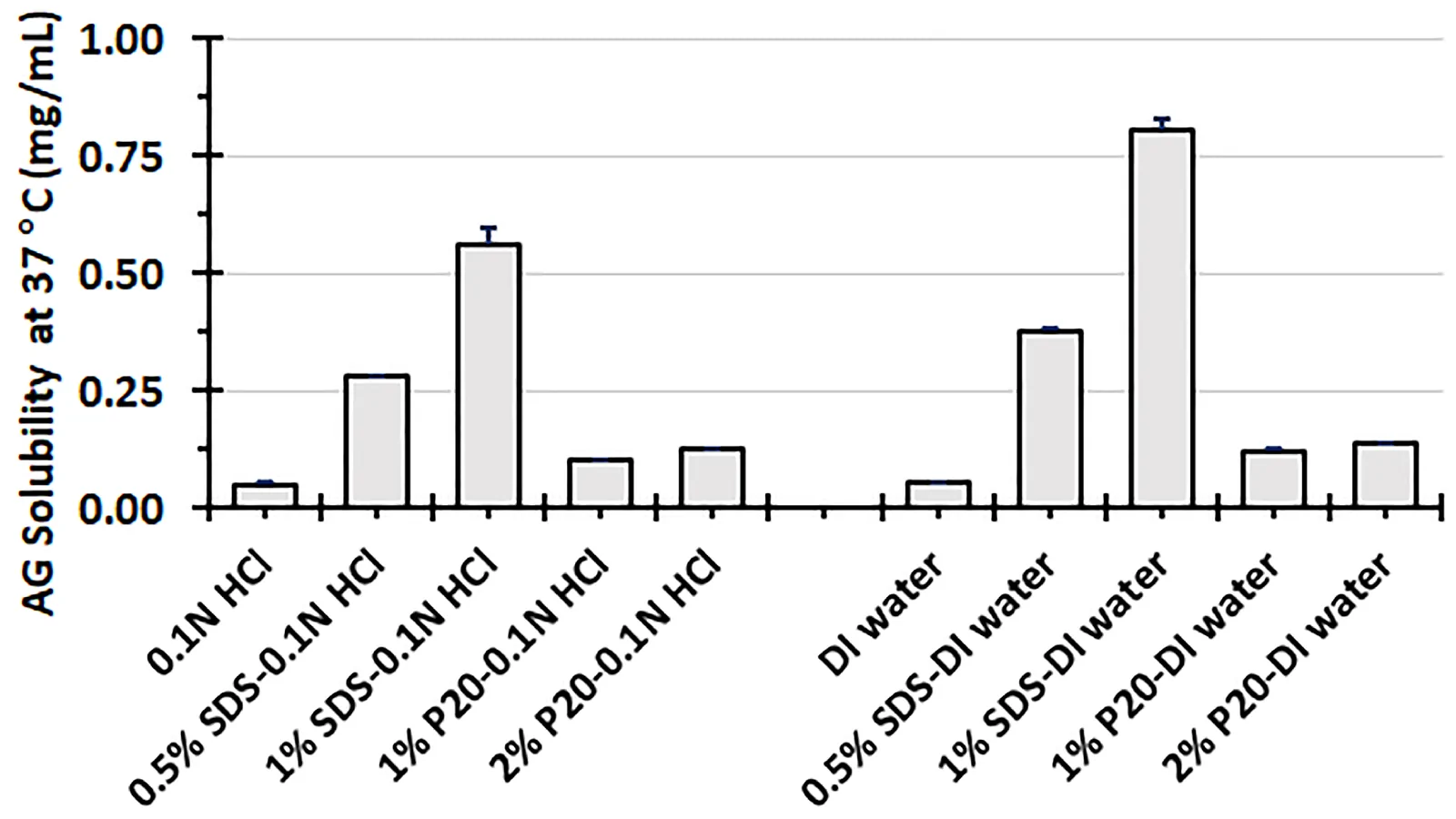
Solubility (37 °C) of AG in aqueous medium, deionized water, and 0.1 N HCl (pH 1.1), with and without solubilizers (SDS and P20). Data are expressed as mean ± SD (*n* = 3). Abbreviations: AG—andrographolide; DI water—deionized water; HCl—hydrochloric acid; P20—polysorbate 20; N—normality; SDS—sodium dodecyl sulfate.

Dissolution evaluation is vital for assessing the release characteristics of oral formulations. The solubility of the desired compound in the medium is essential for determining the sink state under the dissolution conditions. Figure 7 illustrates the solubility values of AG compounds in an aqueous medium at 37 °C, which is the physiological temperature. pAG was practically insoluble in deionized water and 0.1 N HCl (pH 1.2, simulated gastric fluid), with a solubility of approximately 0.05 mg/mL. The limited solubility of AG in aqueous media is attributed to its lipophilic nature, as indicated by its experimental log *p* value of 2.63 [3]. The incorporation of solubilizers, specifically 0.5–1% *w*/*v* SDS (anionic surfactant) and 1–2% *w*/*v* P20 (nonionic surfactant), significantly enhanced AG solubility. This phenomenon resulted from the micellar solubilization effect of the surfactants. In a medium containing 0.5–1% *w*/*v* SDS, the solubility of AG ranged from 0.28 to 0.56 mg/mL in 0.1 N HCl and from 0.38 to 0.80 mg/mL in deionized water. A smaller enhancement in solubility was observed for P20. The solubility of AG in a 1–2% *w*/*v* P20 medium was measured to be 0.10–0.13 mg/mL in 0.1 N HCl and 0.12–0.14 mg/mL in deionized water. The reduced solubility of AG in 0.1 N HCl may be associated with its degradation at acidic pH. Research has indicated that AG is subject to destruction by hydrolysis in strongly acidic environments (pH 1–3) or in the presence of HCl, resulting in the formation of various degradation products [12,94].

An in vitro study was performed to examine the dissolution of AG from pAG and APEs, along with their PM and LS forms. The paddle method and USP apparatus II were used in this investigation. The dissolution profiles were compared using a mathematical calculation for the *f*_2_ value. It should be noted that the *f*_2_ methodology is a recognized technique for demonstrating dissolution similarity. It is preferred because of its relative ease of use, straightforward calculation, and because it allows for the establishment of a clear acceptance criterion for profile similarity (i.e., *f*_2_ ≥ 50). An *f*_2_ value of 50 signifies a mean discrepancy of 10% throughout the designated period [95].

To accurately evaluate the dissolution characteristics of AG, 0.1 N HCl which has a pH of 1.1 ± 0.1 was used to simulate a stomach fluid. For immediate-release preparations, 0.1 N HCl or simulated gastric fluid without enzymes serves as the standard dissolution medium [96]. The selected dissolution medium for conducting in vitro dissolution studies of formulations must be sufficiently discriminative enough to distinguish between release patterns and assist in identifying the formulation variables that influence the release of active molecules from the preparations during the early stages of product development. This study examined dissolution under both non-sink and sink conditions. Taking into account the solubility of the AG compound (C_s_), amount of AG in the dissolution test sample (dose, 30 mg), and volume of the dissolution medium (V, 600 mL), the sink index was calculated using the formula C_s_/(dose/V). In the case of 0.1 N HCl, the solubility was determined to be 0.050 mg/mL, resulting in a sink index of 1.0, which indicates intermediate non-sink conditions [97,98,99]. Non-sink conditions (intermediate) were chosen due to their capacity to distinguish between dissolution profiles of various formulations. The non-sink conditions are crucial for assessing the actual efficacy of supersaturating formulations and preparations [97,100]. Prior studies on drug delivery systems for AG have concentrated on the dissolution of AG under non-sink conditions (sink index less than 3). This encompasses research on AG–2-hydroxypropyl-β-cyclodextrin complexes [24], nanosuspensions, dried nanocrystal particles, solid self-nanodispersion delivery systems [83], liquisolid systems [28,29], and AG nanocrystals [101].

For 0.1 N HCl with 0.5% SDS, the solubility value of AG was determined to be 0.279 mg/mL. Consequently, the sink index was calculated to be 5.6, indicating perfect sink conditions. It is well established that incorporating SDS into dissolution media can uphold sink conditions and facilitate the dissolution of immediate-release preparations. SDS comprising 0.1 N HCl has been extensively utilized in previous dissolution studies of various hydrophobic drug preparations as well as AG or Andrographis formulations [14,22,28,102].

**Figure 8:**
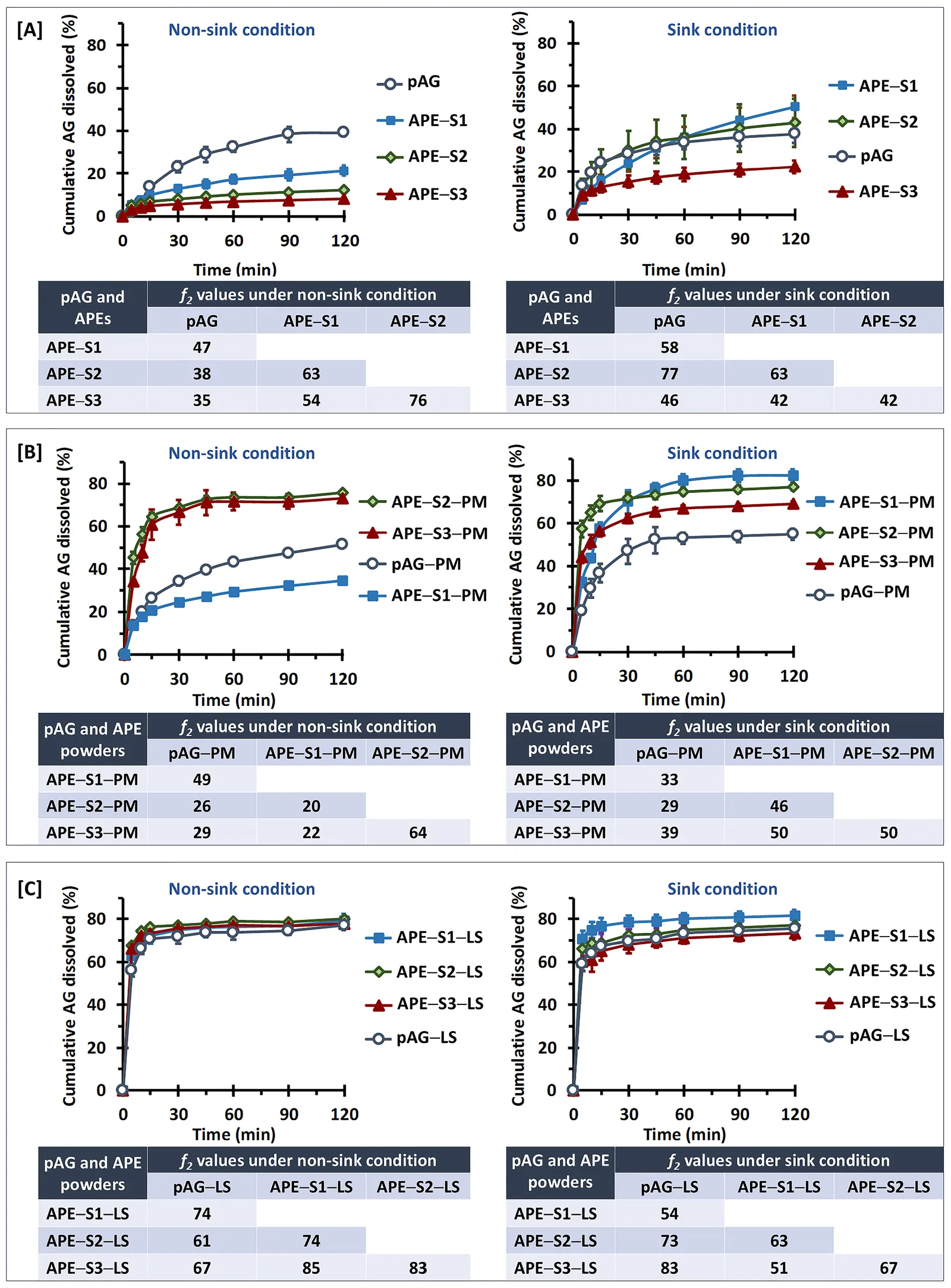
Dissolution of AG molecules under non-sink (**left**) and sink (**right**) conditions, along with the *f*_2_ values of pAG and APEs (**A**), pAG–PM and APE–PMs (**B**), as well as pAG–LS and APE–LS (**C**). Data are expressed as mean ± SD (*n* = 6). Abbreviations: AG—andrographolide; APE—*Andrographis paniculata* extract; APE–S1—*Andrographis paniculata* extract derived from source 1; APE–S2—*Andrographis paniculata* extract derived from source 2; APE–S3—*Andrographis paniculata* extract derived from source 3; *f*_2_—similarity factor; LS—liquisolid; pAG—pure andrographolide powder; PM—physical mixture.

Figure 8A illustrates that the dissolution behaviors of the AG compound from pAG and various APEs were constrained under both non-sink and sink conditions. Under non-sink conditions, the dissolution of AG molecules from APEs significantly differed from that of pAG, as evidenced by *f*_2_ values below 50. The APEs from the three sources demonstrated similar patterns of AG dissolution (*f*_2_ > 50). This finding may have resulted from the very low dissolution of AG from APEs. The APEs exhibited significant hydrophobicity. Upon exposure to the dissolution medium, the extract formed aggregates at the bottom of the dissolution vessel. This resulted in a restricted surface area that is accessible for dissolution for the APEs. The dissolution parameters presented in Table 3 demonstrate the superior dissolution of AG, both in terms of percentage dissolved or cumulative dissolved amount within 5 min (Q_5min_) and dissolution efficiency at 120 min (DE_120min_), from pAG or APEs under sink compared to non-sink conditions. This is attributed to the enhanced solubility of the AG molecule and the superior wettability of the crude extract in a 0.5% SDS−0.1 N HCl medium. Nevertheless, the Q_5min_ and DE_120min_ values for AG dissolution from pAG and APEs in the sink state ranged from 6.7% to 13.3% and 17.6% to 33.6%, respectively.

The limited dissolution of AG from pAG and APEs is consistent with previous studies [14,22,28]. This is attributed to the hydrophobicity and poor water solubility of AG [3]. Furthermore, the hydrophobic nature of crude APEs may may contribute to inadequate wettability and AG dissolution.

Figure 8B depicts the AG dissolution from pAG–PM and APE–PMs. The improvement in AG dissolution when it was physically mixed with hydrophilic excipients, including MCC and CSD particles, was evident. The potential adsorption of APEs onto the porous structure of the excipients (Figure 5) may also play a role in the improved dissolution of AG. Furthermore, the pulverization of APEs with MCC and CSD enhanced the accessible surface area for the dissolution medium. Compared to the crude extract, APE–S2–PM and APE–S3–PM demonstrated significantly improved AG dissolution percentage and efficiency, achieving a DE_120min_ value of 63.1–71.6% under sink conditions. For APE–S2–PM and APE–S3–PM, the Q_5min_ values exhibited enhancements of 9–11 times and 4–5 times, respectively; conversely, the DE_120min_ values demonstrated improvements of 7–10 times and 2–4 times under non-sink and sink settings, respectively.

Notably, the dissolution of both APE–S2–PM and APE–S3–PM under non-sink conditions was similar to that observed under sink conditions. Additionally, APE–S2–PM and APE–S3–PM exhibited comparable dissolution behaviors. This indicates that APE–S2–PM and APE–S3–PM were unconstrained. This implies that simply dispersing APE–S2 and APE–S3 crude extracts with hydrophilic excipients (MCC and CSD) to enhance wettability and increase the available surface area is an effective approach for enhancing AG dissolution.

In contrast, limited improvement in AG dissolution was observed with pAG–PM and APE-S1–PM. Their DE_120min_ values improved by only 1–2 times under non-sink and sink conditions, compared to pAG or APE–S1. It was also observed that pAG–PM exhibited different AG dissolution compared to all APE–PMs under both non-sink and sink conditions (*f*_2_ < 50). Similarly, the dissolution characteristics of APE–S1–PM under non-sink conditions differed from those of the other APE–PMs (*f*_2_ < 50). This suggests that the physical dispersion of pAG and APE–S1 with hydrophilic excipients was not a successful at enhancing AG dissolution. This could be attributable to the crystalline properties of pAG–PM and APE–S1–PM, in contrast to APE–S2–PM and APE–S3–PM (Figure 5).

Notably, the variation in AG dissolution from the different APE–PMs obtained in this study aligns with prior reports. Rangkadilok et al. [14] reported that the dissolution of AG compounds from four commercially available Andrographis extract capsules in a 0.1 N HCl medium during the initial 15 min ranged from less than 10% to approximately 55%. Under sink conditions (0.2% SDS in 0.1 N HCl), the cumulative dissolution of AG at 15 min also ranged from less than 10% to approximately 55%.

The dissolution of AG from LS powders is illustrated in Figure 8C. It is clearly seen that the dissolution of AG from pAG–LS and the three APE–LSs was similar (*f*_2_ > 50) under both non-sink and sink conditions. A significant improvement in the dissolution percentage and efficiency was observed. Their Q_5min_ values ranged between 56.1–67.9% and 59.1–70.6%, alongside DE_120min_ values ranging between 71.3–76.2% and 68.9–77.6% under both non-sink and sink conditions.

The similarity in AG dissolution, irrespective of the AG compound source, with a maximum dissolution plateau achieved within 15 min, signifies the decrease in the source-dependent variability in dissolution of AG via LS powders. The enhancement in the dissolution of AG from AG–LS and APE–LSs aligns with prior research examining the enhancement of dissolution through LS systems for several phytopharmaceuticals, such as diarylheptanoids, curcumin, and cannabinoids [28,29,31,34,35,36]. Previous reports have highlighted that LS technology can enhance the dissolution of the loaded active compound via various mechanisms. The primary mechanisms include the conversion of into a molecularly dispersed state, the enhancement of the available surface area for dissolution due to deposition on the surface area of the carrier-coating materials, as well as improved wettability and increased apparent solubility induced by nonvolatile liquid vehicles [16,29,35]. Nevertheless, the greater dissolution of AG from the LS systems compared to that from the PM may be due to the greater dilution of AG in the excipients, as different ratios of AG-to-MCC/CSD were observed between the PM and LS systems.

The similarity in the AG dissolution between pAG-LS and APE-LSs was hypothesized to be primarily caused by the effect of solid-state transformation induced by the liquid vehicle, with the fraction of molecularly dispersed AG in LS powder being equal to 1. However, this study primarily focused on AG compounds. APEs also contain other diterpene lactones, particularly DDAG. Future investigations should study the solubility, solid state, dissolution behaviors, and stability of LS powders of pure DDAG as well as other focused diterpene lactones. This issue is significant because of the potential transformation of AG into DDAG during storage [103]. Additionally, to further confirm the similarity between the formulations, the mean dissolution values of 12 replicates will be used.

### 3.4. Stability of pAG, APEs, and Their PM and LS Powders

The preliminary study regarding physical and chemical stabilities of the AG molecules in APEs, APE–PMs, and APE–LS powders were compared with those of pAG, pAG–PM, and pAG–LS powders. The tested specimens were securely sealed in aluminum pouches and stored for 3 months under three distinct conditions: 5 ± 3 °C (refrigerated storage), 30 °C/75% RH (long-term storage), and 40 °C/75% RH (accelerated aging) [50].

#### 3.4.1. Appearance and Flowability

**Figure 9:**
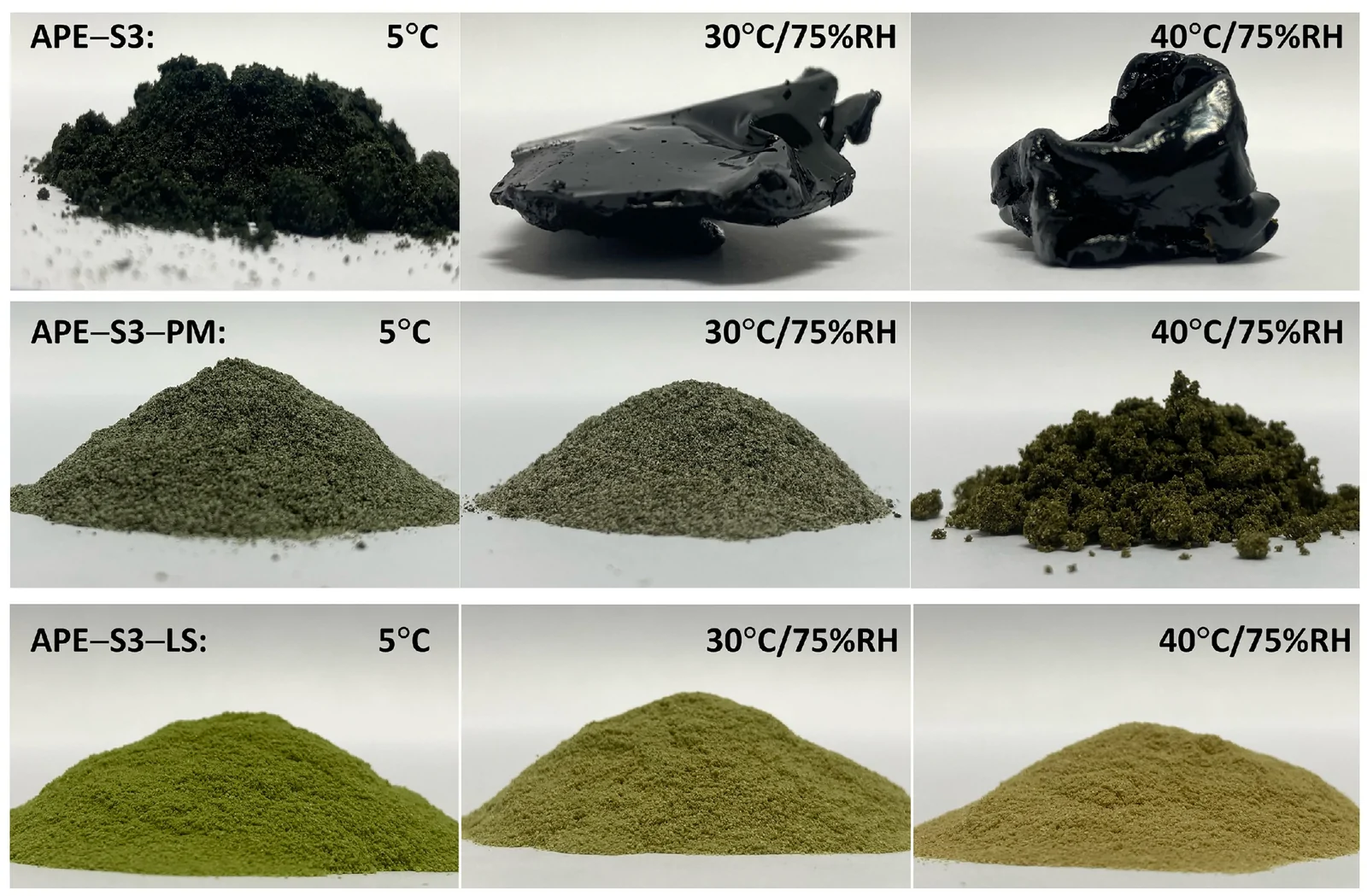
Physical appearance of aged APE–S3 and its powder in the form of APE–S3–PM and APE–S3–LS after being stored under different conditions for 3 months. Abbreviations: APE–S3—*Andrographis paniculata* extract derived from source 3; LS—liquisolid; PM—physical mixture; RH—relative humidity.

The appearance of pAG, pAG–PM, and pAG–LS remained unchanged after three months of storage, preserving the characteristics of their freshly formed state (Figure 3). The flowability, as indicated by Carr’s index values, of the 3-month-old pAG–PM and pAG–LS remained good. For APEs, melting and fusion of aged samples, irrespective of the source, were observed under long-term and accelerated-storage conditions (Figure 9). The 3-month-old APE–PM exhibited adhesive aggregation, particularly under accelerated aging conditions. This may have resulted from the liquefaction of the constituents of the crude extract. A slight change in the color of the APE–PM subjected to accelerated aging to a brown shade was observed. Nonetheless, the physical state and dark green hue of APEs and APE–PMs stored under refrigerated conditions remained similar to their original characteristics.

All aged APE–LS samples, irrespective of the storage conditions, were dry and exhibited free-flowing characteristics (Figure 9). Their flowability, as determined by Carr’s index, ranged from fair to good. Notably, a distinct change in color to a yellow–brown hue was observed in the APE–LSs from all APE sources after accelerated aging. The preservation of the appropriate physical characteristics of LS systems is consistent with previous studies. It is crucial to note that physical stability is not a significant issue for LS systems. The liquid component and carrier-coating material establish a physically stable system that is preserved over time. Numerous studies have established that LS systems exhibit sufficient storage stability under various conditions [35,36,69,104,105].

#### 3.4.2. Solid-State Characteristics

**Figure 10:**
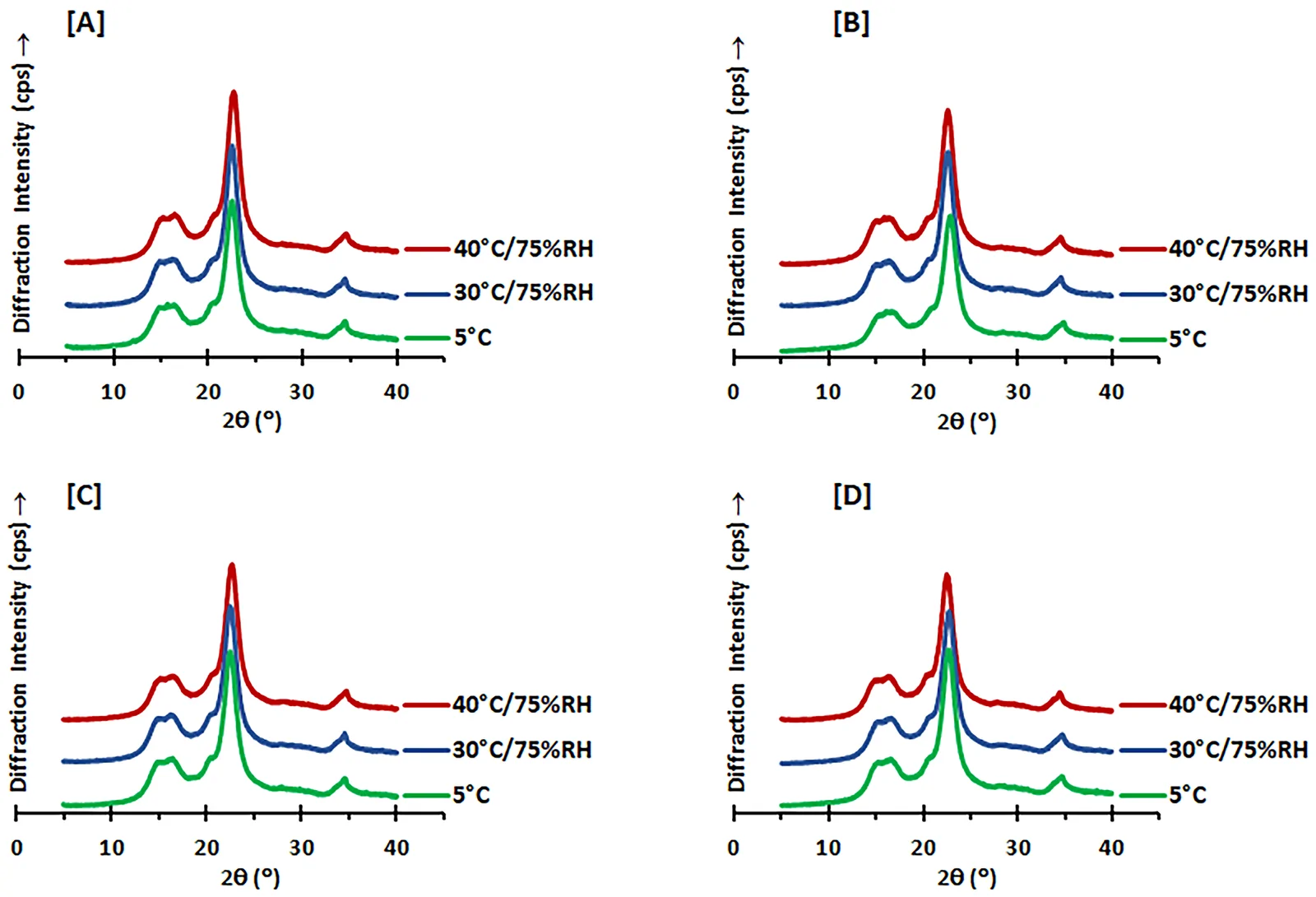
PXRD patterns of pAG–LS (**A**), APE–S1–LS (**B**), APE–S2–LS (**C**), and APE–S3–LS (**D**) after three months of storage under 3 different conditions. The arrow indicates the diffraction intensity direction. Abbreviations: APE–S1—*Andrographis paniculata* extract derived from source 1; APE–S2—*Andrographis paniculata* extract derived from source 2; APE–S3—*Andrographis paniculata* extract derived from source 3; LS—liquisolid; pAG—pure andrographolide powder; RH—relative humidity.

Figure 10A–D displays the PXRD patterns of the three-month-old LS powders. It is evident that the AG−LS and APE−LS powders preserved their PXRD patterns, showing patterns similar to those of their fresh counterparts (Figure 4). These results suggest that there was no crystallite development after three months of storage, irrespective of the storage conditions. This aligns with other studies indicating that aging does not influence the PXRD patterns of LS systems after storage. These investigations utilized non-volatile liquid vehicles with sufficient solvent capabilities for active compounds [29,31]. Previous research has indicated that recrystallization of AG after three months of storage occurred when the molecularly dispersed drug fraction parameter was significantly below 1, failing to preserve its non-crystalline form over the evaluated storage duration [29]. Nevertheless, as the residual liquid vehicle remaining in the LS systems could alter both the dissolution behavior and physical characteristics of the formulation, it is suggested that the residual liquid vehicle, including NMP for the AG-LS and APE-LSs in this study, should be measured after storage. Moreover, the physical stability and transformation of pAG-LS and APE-LSs after storage will be further assessed, including thermal behavior changes using differential scanning calorimetry, FTIR spectra alterations, moisture content changes, and morphological changes using SEM.

#### 3.4.3. Percentage of AG Remaining

**Figure 11:**
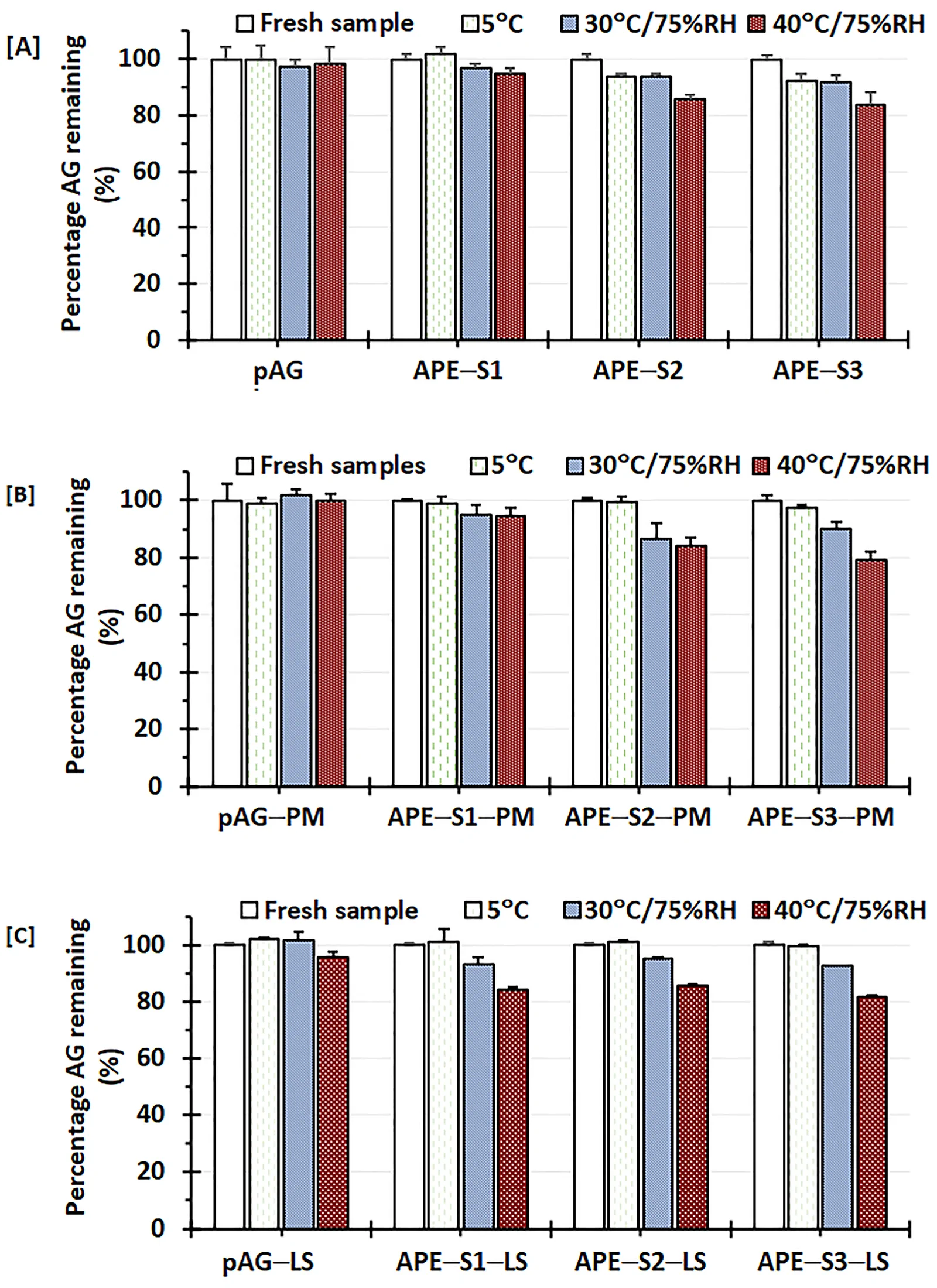
Percentage of AG remaining in samples after three months of storage under various conditions compared to fresh samples: pAG and APEs (**A**), pAG–PM and APE–PMs (**B**), and pAG–LS and APE–LSs (**C**). Data are expressed as mean ± SD (*n* = 6). Abbreviations: AG—andrographolide; APEs—*Andrographis paniculata* extracts; APE–S1—*Andrographis paniculata* extract derived from source 1; APE–S2—*Andrographis paniculata* extract derived from source 2; APE–S3—*Andrographis paniculata* extract derived from source 3; LS—liquisolid; pAG—pure andrographolide powder; PM—physical mixture; RH—relative humidity.

Figure 11A illustrates the percentage of the AG component remaining in the APEs compared with pAG. pAG exhibited remarkable stability, regardless of the storage conditions. The percentage of remaining AG in the 3-month-old pAG decreased insignificantly from 97.3 ± 2.6% to 99.9 ± 4.8% (*p* > 0.05). The amount of AG remaining in the aged APEs varied based on the source. In APE–S1, a notable decrease in AG was only observed in specimens stored under long-term storage and accelerated aging conditions, with AG levels ranging from 96.8 ± 1.6% to 95.0 ± 2.1% (*p* < 0.05). The remaining percentage of the AG compound in 3-month-old APE–S2 and APE–S3 decreased significantly compared to that in the fresh specimens (*p* < 0.05). Substantial AG degradation in these APEs when stored under accelerated aging conditions was noted, with values between 83.6 ± 4.5% and 85.8 ± 1.6%.

The percentage of AG remaining in APE–PMs after three months of storage, compared to pAG–PM, is illustrated in Figure 11B. Similar to pAG, pAG–PM exhibited good AG compound stability, with minimal changes observed in aged specimens compared to fresh specimens, irrespective of the storage conditions (*p* > 0.05). For all APE–PMs, a slight decrease in AG was observed in samples aged under refrigerated conditions (*p* > 0.05), whereas long-term storage and, in particular, accelerated aging conditions led to a considerable decrease in AG content (*p* < 0.05). Interestingly, notable differences in the remaining AG under accelerated conditions were observed as follows: pAG–PM > APE–S1–PM > APE–S2–PM > APE–S3–PM (*p* < 0.05). The percent AG retained in pAG, APEs, pAG–PM, and APE–PMs under refrigerated conditions was comparable (*p* > 0.05).

Figure 11C shows the percentage of AG remaining in LS powders after three months of storage. All LS powders aged under refrigerated conditions were stable, with negligible changes in the remaining AG content (*p* > 0.05). The percent AG remaining following long-term and accelerated aging storage conditions for APE–LSs was comparable to that of APE–PMs. A substantial effect of AG degradation at elevated temperatures during accelerated conditioning was observed for both pAG–LS and APE–LSs (*p* < 0.05).

The greater reduction in the remaining AG content in pAG–LS relative to that in pAG–PM may be attributed to the transformation of the solid-state of AG molecules into a non-crystalline form owing to the treatment with LS technology. A similar trend was observed, with a greater decrease in the remaining AG percentage in APE–S1–LS than in APE–S1–PM. Furthermore, the smaller percentage of remaining AG under accelerated conditions for all test samples clearly underscores the impact of storage temperature. This is consistent with previous studies [103,106,107,108]. According to Lomlim et al. and Plubrukarn et al., AG undergoes degradation when stored at elevated temperatures (e.g., 45–70 °C), with the extent of degradation increasing with increasing temperatures. Under heat-accelerated conditions, crystalline AG demonstrated great stability for up to three months at 70 °C/75% RH. In contrast, AG in the amorphous form decomposed rapidly at this elevated temperature, yielding the primary degradation product—DDAG. The degradation of AG from pAG and powdered Andrographis derived from the aerial parts of *A. paniculata* under elevated temperature conditions followed second-order kinetics. The estimated rate constant at 25 °C, calculated using the Arrhenius methodology, was found to be 3.8–6.6 × 10^−6^ d^−1^ [107,108]. Because of their temperature-sensitive degradation, preparations containing pAG and APEs, especially in the form of LS powder, are categorized as “drug products requiring storage under refrigeration.” The appropriate storage temperature for these products is 5 ± 3 °C [50].

The degradation of AG molecules has been reported to involve hydrolysis, particularly at the lactone ring, leading to the opening of the lactone ring and the formation of more polar and water-soluble degradation products. Elevated temperatures facilitate the hydrolytic cleavage of lactone rings [107,109]. Rattanaya et al. recently reported on the storage stability of diterpene lactones in aged *A. paniculata* extracts. This report indicates that AG compounds degrade at slow rates and are presumably transformed into DDAG through dehydration [103].

#### 3.4.4. Dissolution Behaviors of AG

**Figure 12:**
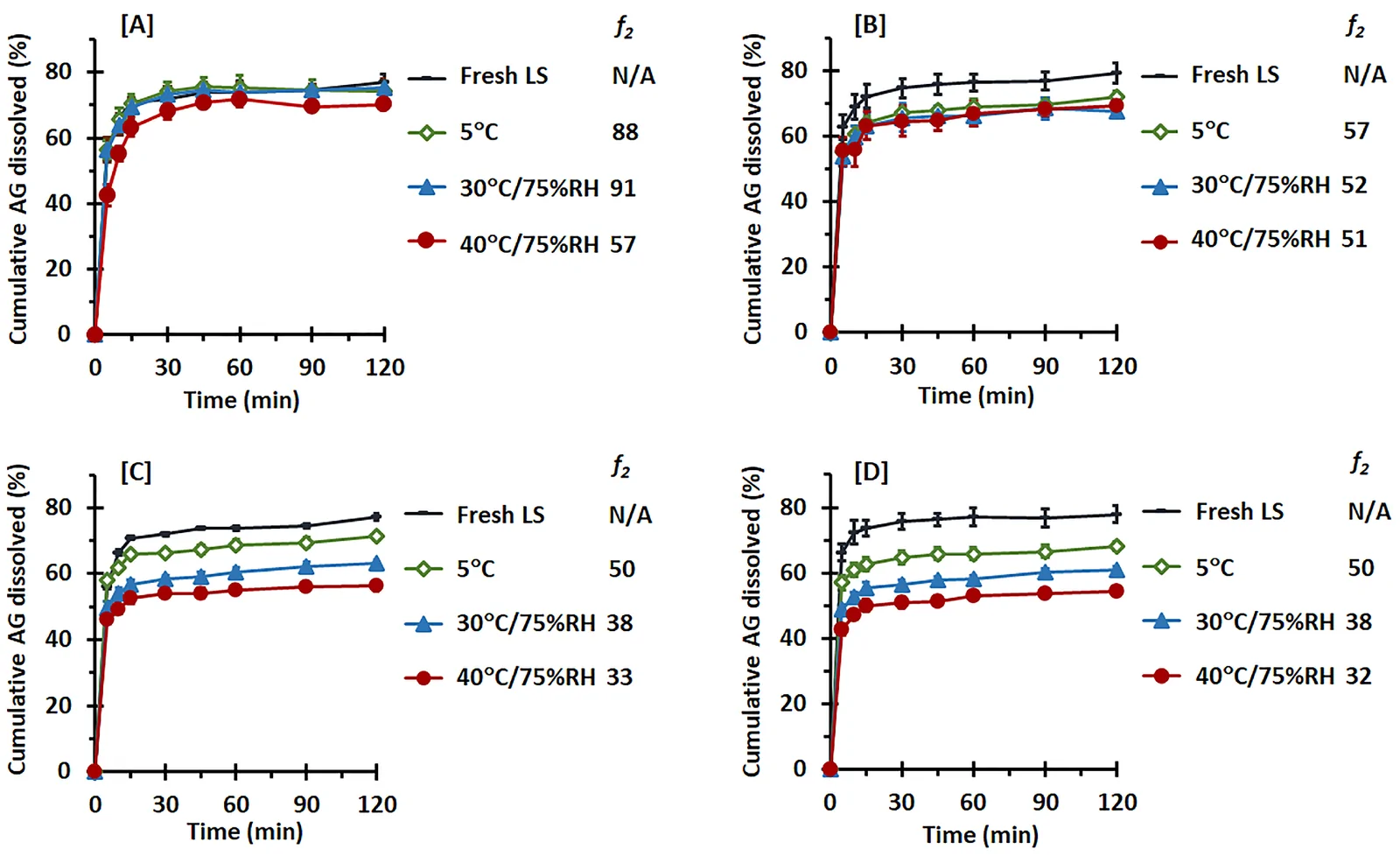
Dissolution profiles and *f*_2_ values of AG from 3-month-old pAG–LS and APE–LSs stored under different conditions compared with fresh specimens under non-sink conditions: pAG–LS (**A**), APE–S1–LS (**B**), APE–S2–LS (**C**), and APE–S3–LS (**D**). Data are expressed as mean ± SD (*n* = 6). Abbreviations: AG—andrographolide; APE–S1—*Andrographis paniculata* extract derived from source 1; APE–S2—*Andrographis paniculata* extract derived from source 2; APE–S3—*Andrographis paniculata* extract derived from source 3; *f*_2_—similarity factor; LS—liquisolid; N/A—not applicable; pAG—pure andrographolide powder; PM—physical mixture; RH—relative humidity.

After observing the enhanced physical stability of LS powders during storage, the dissolution characteristics of AG in 3-month-old LS powders were examined. Figure 12 demonstrates the impact of aging on the dissolution of AG in LS powders under non-sink conditions. It should be noted that the percentage of AG dissolved in aged samples was calculated in relation to the amount present in the fresh samples. The dissolution of AG from aged pAG–LS (Figure 11A) was similar to that of fresh powders (*f*_2_ > 50), regardless of the storage conditions. Likewise, all APE–LS powders stored under refrigerated conditions demonstrated AG solubility comparable to that of fresh specimens (*f*_2_ ≥ 50). Differences were observed across the APE–LS powders from different sources after being subjected to both long-term storage and accelerated aging conditions. The aged APE–S1–LS demonstrated dissolution comparable to that of the fresh samples (*f*_2_ > 50).

Several studies have demonstrated that aging does not affect the characteristics and dissolution patterns of LS systems [31,35,37,41,58,69,110,111]. Nevertheless, a notable dissimilarity in AG dissolution from fresh samples was detected in aged APE–S2–LS and aged APE–S3–LS under harsh conditions (*f*_2_ < 50). An *f*_2_ value of less than 50 may indicate an average discrepancy in AG dissolution of over 10% at all designated time points. This can be attributed to a decrease in the remaining AG, resulting in a diminished quantity of AG available for dissolution compared to that in the fresh powder. Multiple studies have demonstrated that aging has a minor influence on the dissolution of these systems, particularly when using MCC and CSD as carriers and coating materials [35,36,69,104,105,110].

## 4. Conclusions

This study performed the first application of LS technology in the fabrication of crude extract powders with enhanced and reduced variability in dissolution behaviors of AG compound, a phytopharmaceutical marker of APEs. LS formulations of pAG and APEs were successfully produced, which exhibited remarkable physical appearances and acceptable flow properties. Solid-state investigations revealed that the APEs exhibited different crystallinity patterns. This may cause variations and restrict the dissolution of the AG compounds. The LS systems, on the other hand, exhibited the non-crystalline state of the AG molecule, leading to an improvement and reduction in the variability in AG dissolution from formulations derived from diverse Andrographis sources. Preliminary stability studies conclusively demonstrated that all formulated LS powders did not exhibit any marked changes in physical appearance, flowability, or solid state, regardless of the storage conditions. The findings of this study provide essential insights into the use of LS technology as an effective pharmaceutical approach for manufacturing herbal extract powders with superior physical characteristics and stability, as well as improved and assimilated dissolution of bioactive markers. Nevertheless, this study focused exclusively on AG molecules. Additional research on other diterpene lactones, such as DDAG, along with other phytopharmaceuticals from different herbal extracts may be necessary to confirm the efficacy of LS technology in reducing source-dependent variability in the dissolution of herbal extracts.

Significantly, this study employed NMP as a model liquid vehicle in LS systems to clarify the solvation prerequisites for obtaining a complete fraction of molecular dispersion, thereby minimizing the inconsistency in dissolution of a bioactive marker from various herbal extract sources. In terms of NMP-based LS systems for oral application, extensive preclinical toxicological assessments under conditions of repeated or long-term exposure, such as sub-chronic and chronic toxicity, are essential to verify their clinical translation. For APE-LS systems, further research should focus on identifying an orally acceptable solvent system that offers similar AG dissolution improvements while remaining below the recognized regulatory safety thresholds. Safer solvent systems that can replicate the essential solvation characteristics of NMP must be systematically devised. Such systems can be formulated using binary or ternary co-solvent blends, hydrotrope–cosolvent pairings, or pharmaceutically acceptable deep eutectic formulations.

## Figures and Tables

**Table 1:** Compositions of LS and PM powders of pAG and APEs and estimated ratio of AG solubility in NMP to AG concentration in liquid medication.

pAG or APE Powder	Composition (%*w*/*w*)				pAG or APE Concentration in Liquid Medication (% *w*/*w*) *	AG Concentration in Liquid Medication (C_AG_) ** (% *w*/*w*) *	Estimated S_AG_/C_AG_ Value ***
	pAG or APE	NMP	MCC	CSD			
LS powders							
pAG–LS	2.0	18.3	75.9	3.8	10	9.97	3.3
APE–S1–LS	8.1	12.2	75.9	3.8	40	9.64	3.4
APE–S2–LS	8.1	12.2	75.9	3.8	40	6.84	4.9
APE–S3–LS	8.1	12.2	75.9	3.8	40	5.96	5.6
PM powders							
pAG–PM	10	0	85.7	4.3	N/A	N/A	N/A
APE–S1–PM	40	0	57.1	2.9	N/A	N/A	N/A
APE–S2–PM	40	0	57.1	2.9	N/A	N/A	N/A
APE–S3–PM	40	0	57.1	2.9	N/A	N/A	N/A

* Percentage (% *w*/*w*) of pAG in the pAG–NMP or APE in the APE–NMP solution. ** AG concentration in liquid medication or C_AG_ was calculated from the AG content in the pAG and APEs: APE-1 (24.1 ± 1.1% AG), APE-2 (17.1 ± 1.4% AG), and APE-3 (14.9 ± 1.2% AG). The AG content of pAG was 99.7 ± 3.5%. *** Estimated S_AG_/C_AG_ or ratio of AG’s solubility in NMP to AG concentration in liquid medication (C_AG_, %*w*/*w*) was obtained by dividing the estimated solubility value in %*w*/*w* (S_AG_) of AG in the liquid vehicle (NMP, 33.2% *w*/*w*) by the AG concentration in liquid medication with the same vehicle [47]. N/A = not applicable. NMP, MCC, and CSD functioned as liquid vehicles, carriers, and coating materials in the LS powder, respectively, with their quantities calculated based on an L_f_ value of 0.255. Abbreviations: APE(s)—*Andrographis paniculata* extract(s); APE–S1—*Andrographis paniculata* extract derived from source 1; APE–S2—*Andrographis paniculata* extract derived from source 2; APE–S3—*Andrographis paniculata* extract derived from source 3; CSD—colloidal silicon dioxide; LS—liquisolid; MCC—microcrystalline cellulose; NMP—N-methyl-2-pyrrolidone; pAG—pure andrographolide powder; PM—physical mixture.

**Table 2:** Flowability and AG content of PM and LS powders of pAG and APEs.

pAG or APE Powder	Carr’s Index ^a^	Flow Property *	Angle of Repose (°) ^a^	Flow Property **	% AG Loading (mg/g) ^b^	
					Theoretical Values	Measure Values
PM powders						
pAG–PM	16.4 ± 1.1	Fair	25.7 ± 0.2	Excellent	19.94	19.40 ± 0.30
APE–S1–PM	9.8 ± 1.3	Excellent	26.7 ± 0.2	Excellent	19.52	18.64 ± 0.31
APE–S2–PM	14.6 ± 1.9	Good	26.6 ± 0.2	Excellent	13.85	13.23 ± 0.15
APE–S3–PM	12.0 ± 2.2	Good	26.6 ± 1.1	Excellent	12.07	11.53 ± 0.19
LS powders						
pAG–LS	13.9 ± 2.0	Good	34.4 ± 0.1	Good	99.70	98.50 ± 0.60
APE–S1–LS	10.6 ± 1.5	Excellent	26.2 ± 0.8	Excellent	96.40	96.69 ± 0.96
APE–S2–LS	10.3 ± 1.6	Good	27.1 ± 0.3	Excellent	68.40	69.63 ± 0.48
APE–S3–LS	14.2 ± 2.5	Good	25.8 ± 1.5	Excellent	59.60	56.86 ± 0.60

Data are expressed as mean ± SD, ^a^ *n* = 3, ^b^ *n* = 6. Flowability is classified based on the value of * Carr’s index and ** angle of repose, according to the USP [48]. Abbreviations: APE(s)—*Andrographis paniculata* extract(s); APE–S1—*Andrographis paniculata* extract derived from source 1; APE–S2—*Andrographis paniculata* extract derived from source 2; APE–S3—*Andrographis paniculata* extract derived from source 3; LS—liquisolid; pAG—pure andrographolide powder; PM—physical mixture.

**Table 3:** In vitro dissolution parameters of AG dissolution from pAG and APEs, along with their PM and LS powders, under non-sink and sink conditions.

pAG or APE Powder	Non-Sink Condition		Sink Condition	
	Q_5min_ (%)	DE_120min_ (%)	Q_5min_ (%)	DE_120min_ (%)
pAG and APEs				
pAG	1.7 ± 0.1 ^a^	28.6 ± 1.6 ^a^	13.3 ± 1.1 ^a^	30.9 ± 2.8 ^a^
APE–S1	5.5 ± 0.8 ^b^	15.7 ± 1.9 ^b^	6.7 ± 0.7 ^b^	33.1 ± 4.4 ^a^
APE–S2	5.0 ± 0.5 ^b^	9.5 ± 0.6 ^c^	13.2 ± 3.1 ^a^	33.6 ± 8.0 ^a^
APE–S3	3.1 ± 0.4 ^b^	6.4 ± 0.8 ^c^	8.9 ± 1.9 ^b^	17.6 ± 2.8 ^b^
PM powders				
pAG–PM	13.7 ± 0.3 ^c^	39.2 ± 0.9 ^d^	18.9 ± 1.5 ^a^	47.3 ± 3.2 ^c^
APE–S1–PM	13.6 ± 1.1 ^c^	27.4 ± 0.6 ^a^	32.4 ± 2.2 ^c^	71.9 ± 2.4 ^d^
APE–S2–PM	45.3 ± 2.8 ^d^	68.8 ± 1.1 ^e^	57.3 ± 4.1 ^d^	71.6 ± 2.1 ^d^
APE–S3–PM	34.2 ± 0.7 ^e^	65.3 ± 2.8 ^f^	44.0 ± 2.6 ^e^	63.1 ± 1.5 ^e^
LS powders				
pAG–LS	56.1 ± 2.9 ^f^	71.3 ± 1.6 ^e^	59.1 ± 3.0 ^d^	70.1 ± 1.2 ^f^
APE–S1–LS	63.0 ± 3.8 ^g^	73.9 ± 2.8 ^g^	70.6 ± 3.8 ^f^	77.6 ± 2.8 ^d^
APE–S2–LS	67.9 ± 0.7 ^h^	76.2 ± 0.5 ^g^	66.4 ± 3.5 ^f^	72.4 ± 2.3 ^d^
APE–S3–LS	66.1 ± 2.6 ^h^	74.3 ± 2.3 ^g^	60.5 ± 4.7 ^d^	68.9 ± 2.8 ^f^

Data are expressed as mean ± SD (*n* = 6). ^a–h^ Values sharing the same superscript letter are not significantly different (*p* < 0.05), as determined by one-way ANOVA followed by Tukey’s post hoc test. Abbreviations: AG—andrographolide; APE(s)—*Andrographis paniculata* extract(s); APE–S1—*Andrographis paniculata* extract derived from source 1; APE–S2—*Andrographis paniculata* extract derived from source 2; APE–S3—*Andrographis paniculata* extract derived from source 3; DE_120min_—dissolution efficiency at 120 min, which is the percentage of the rectangular area under the dissolution curve up to 120 min; LS—liquisolid; pAG—pure andrographolide powder; PM—physical mixture; Q_5min_ (%)—cumulative dissolved quantity of AG at 5 min.

## Data Availability

The original contributions presented in this study are included in the article/Supplementary Material. Further inquiries can be directed to the corresponding author.

## Supplementary Materials

The following supporting information can be downloaded at: https://www.mdpi.com/article/10.3390/pharmaceutics18080976/s1, Figure S1: The angle of the slide tester [A] and relation between angle of slide and weight ratio of NMP to solid particles (MCC and CSD) together with the flowable liquid retention potential, Φ_ca_ and Φ_co_ (mean ± SD, *n* = 3) [B]; Figure S2: FTIR patterns of pAG, 10% w/w pAG in NMP, and NMP solvent. Abbreviations: NMP–N-methyl-2-pyrrolidone; pAG—pure andrographolide powder; Figure S3: Dissolution of AG molecules under non-sink (left) and sink (right) conditions of pAG [A], APE–S1 [B], APE–S2 [C], and APE–S3 [D]; Figure S4: Dissolution of AG molecules under non-sink (left) and sink (right) conditions of pAG–PM [A], APE–S1–PM [B], APE–S2–PM [C], and APE–S3–PM [D]; Figure S5: Dissolution of AG molecules under non-sink (left) and sink (right) conditions of pAG–LS [A], APE–S1–LS [B], APE–S2–LS [C], and APE–S3–LS [D]; Figure S6: Dissolution of AG molecules under non-sink conditions of 3-month-aged pAG–LS storage under three distinct conditions: 5 ± 3 °C (refrigerated) [A], 30 °C/75% RH (long-term) [B], and 40 °C/75% RH (accelerated) [C]; Figure S7: Dissolution of AG molecules under non-sink conditions of 3-month-aged APE–S1–LS storage under three distinct conditions: 5 ± 3 °C (refrigerated) [A], 30 °C/75% RH (long-term) [B], and 40 °C/75% RH (accelerated) [C]; Figure S8: Dissolution of AG molecules under non-sink conditions of 3-month-aged APE–S2–LS storage under three distinct conditions: 5 ± 3 °C (refrigerated) [A], 30 °C/75% RH (long-term) [B], and 40 °C/75% RH (accelerated) [C]; Figure S9: Dissolution of AG molecules under non-sink conditions of 3-month-aged APE–S3–LS storage under three distinct conditions: 5 ± 3 °C (refrigerated) [A], 30 °C/75% RH (long-term) [B], and 40 °C/75% RH (accelerated) [C]; Table S1: Validation parameters of the HPLC method for AG and DDAG.

## Abbreviations

The following abbreviations are used in this manuscript:

AG	Andrographolide
ANOVA	Analysis of Variance
APE	*Andrographis paniculata* extract
APEs	*Andrographis paniculata* extracts
APE-S1	*Andrographis paniculata* extract derived from source 1
APE-S2	*Andrographis paniculata* extract derived from source 2
APE-S3	*Andrographis paniculata* extract derived from source 3
ATR	Attenuated total reflection
BCS	Biopharmaceutics Classification System
CSD	Colloidal silicon dioxide
DDAG	14-Deoxy-11,12-didehydroandrographolide
DE_120min_	Dissolution efficiency at 120 min
DI water	Deionized water
EtOH	Ethanol (ethyl alcohol)
*f*_2_	Similarity factor
FTIR	Fourier Transform Infrared Spectroscopy
HCl	Hydrochloric acid
HPLC	High-Performance Liquid Chromatography
LD_50_	Median Lethal Dose
L_f_	Liquid Load Factor
LS	Liquisolid
MCC	Microcrystalline cellulose
NMP	N-Methyl-2-pyrrolidone
OECD	Organization for Economic Co-operation and Development
P20	Polysorbate 20
pAG	Pure andrographolide powder
PM	Physical mixture
PXRD	Powder X-ray diffraction
Q_5min_	Percentage (or cumulative amount) of AG dissolved at 5 min
R	Carrier-to-coating material ratio (Q/q) in the liquisolid system
R^2^	Coefficient of determination
RH	Relative humidity
SD	Standard deviation
SDS	Sodium dodecyl sulfate
SEM	Scanning Electron Microscopy
USP	United States Pharmacopeia
