# Supplementary material for: Liquisolid Technology Improves and Reduces Source-Dependent Variability in Andrographolide Dissolution from Andrographis paniculata Extracts
Source: Pharmaceutics. 2026 Aug 8;18(8):976. doi: 10.3390/pharmaceutics18080976 (PMC13516669; doi:10.3390/pharmaceutics18080976)
Supplement: Supplementary file 1 [file pharmaceutics-18-00976-s001.zip › pharmaceutics-4443948-supplementary.pdf]

Supplementary materials

Liquisolid Technology Improves and Reduces Source-Dependent Variability in Andrographolide Dissolution from *Andrographis paniculata* Extracts

Peera Tabboon [1,2], Ekapol Limpongsa [3], Sarunya Tuntiyasawasdikul [1,2], Tanyarat Yodthong [1], Watchara Kanjanakawinkul [4] and Napaphak Jaipakdee [1,2]*

[1] Faculty of Pharmaceutical Sciences, Khon Kaen University, Khon Kaen, 40002 Thailand; peerata@kku.ac.th (P.T.); tanyarat.y@kkumail.com (T.Y.); nj.jaipakdee@gmail.com (N.J.)
[2] Center for Research and Development of Herbal Health Products, Khon Kaen University, Khon Kaen, 40002 Thailand; sarunyat@kku.ac.th (S.T.)
[3] College of Pharmacy, Rangsit University, Pathum Thani, 12000 Thailand; ekapol.l@rsu.ac.th (E.L)
[4] Chulabhorn Royal Pharmaceutical Manufacturing Facilities by Chulabhorn Royal Academy, Chon Buri 20180 Thailand; watchara.kan@cra.ac.th (W.K.)

* Correspondence: nj.jaipakdee@gmail.com; Tel.: +66-81-9749228

[A]

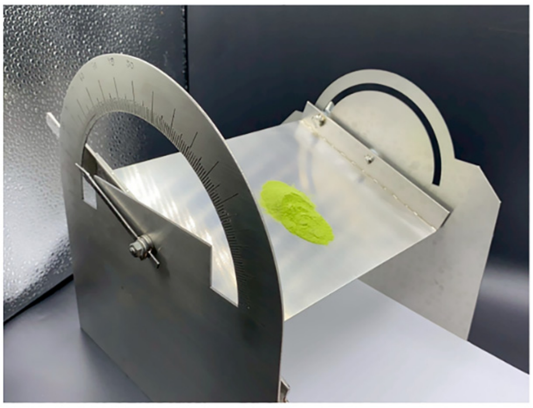

[B]

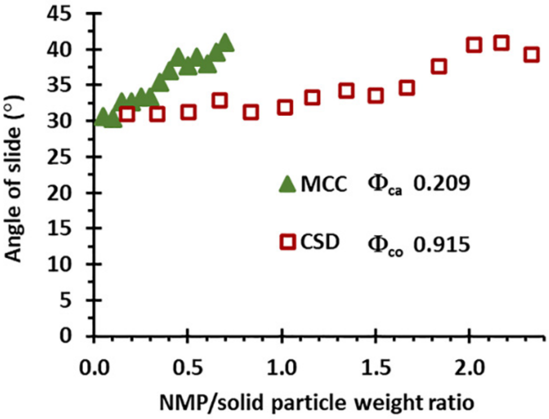

Figure S1. The angle of the slide tester [A] and relation between angle of slide and weight ratio of NMP to solid particles (MCC and CSD) together with the flowable liquid retention potential, Φ_{ca} and Φ_{co} (mean ± SD, n=3) [B].

Table S1. Validation parameters of the HPLC method for AG and DDAG

Parameters	AG	DDAG
Calibration range (μg/mL)	3.125–50	3.125–50
Linearity (R^2)	1.0000	1.0000
LOD (μg/mL)	0.14	0.17
LOQ (μg/mL)	0.45	0.56
Inter-day Precision (%RSD)		
3.125 μg/mL	2.93	2.69
25 μg/mL	2.40	2.46
50 μg/mL	3.97	3.65
Intra-day Precision (%RSD)		
3.125 μg/mL	3.25	3.29
25 μg/mL	1.52	1.56
50 μg/mL	2.19	1.38
Accuracy (%recovery)		
5 μg/mL	100.27 – 104.44	98.15 – 102.37
20 μg/mL	98.22 – 104.55	100.83 – 107.44
40 μg/mL	95.79 – 105.71	98.89 – 103.53

Data are presented as mean ± SD, n = 6 for all cases.

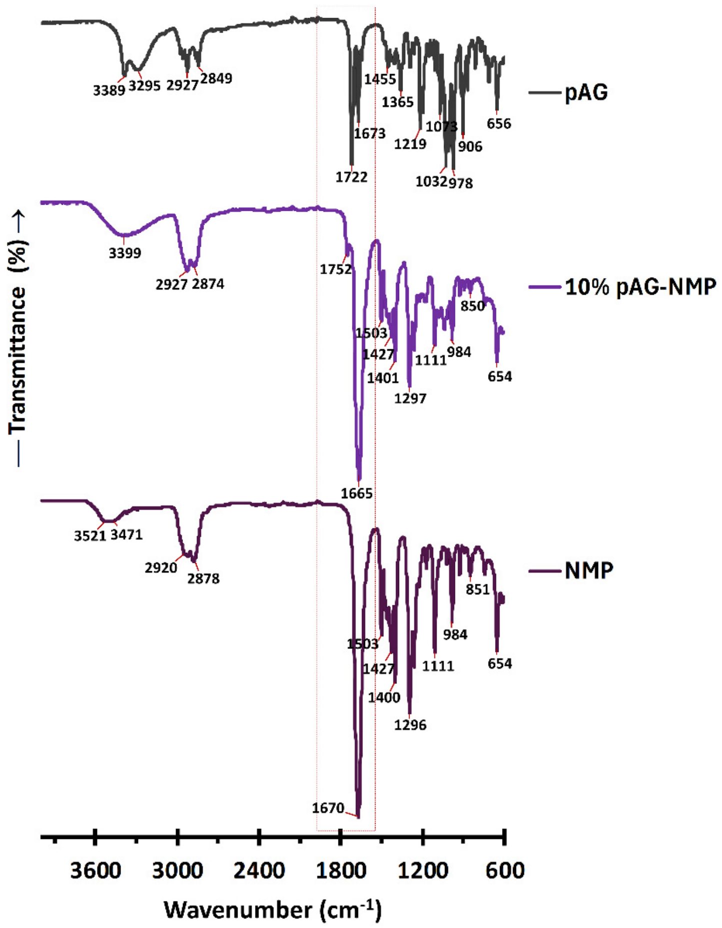

Figure S2. FTIR patterns of pAG, 10% w/w pAG in NMP, and NMP solvent. *Abbreviations*: NMP—N-methyl-2-pyrrolidone; pAG—pure andrographolide powder.

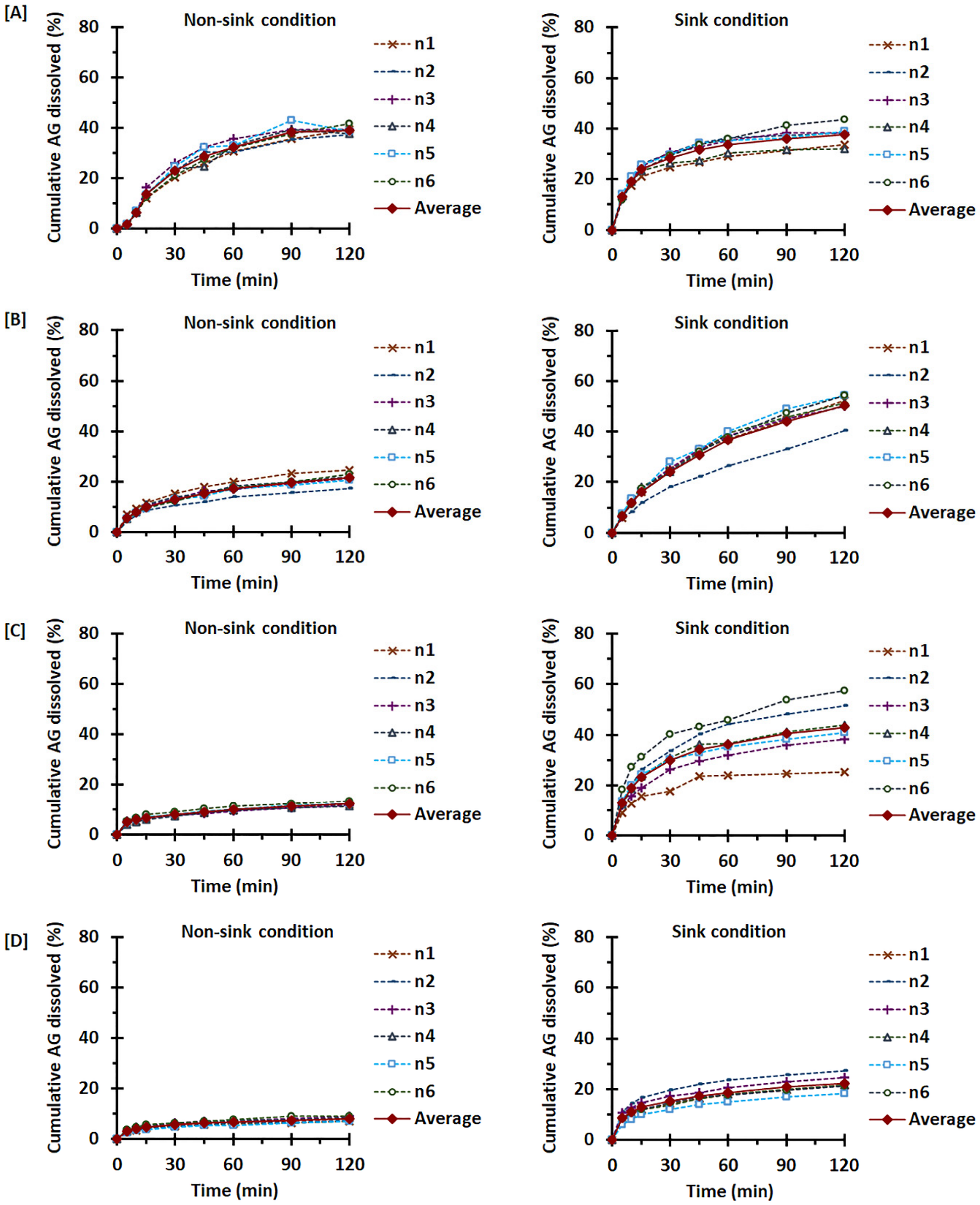

Figure S3. Dissolution of AG molecules under non-sink (left) and sink (right) conditions of pAG [A], APE–S1 [B], APE–S2 [C], and APE–S3 [D].

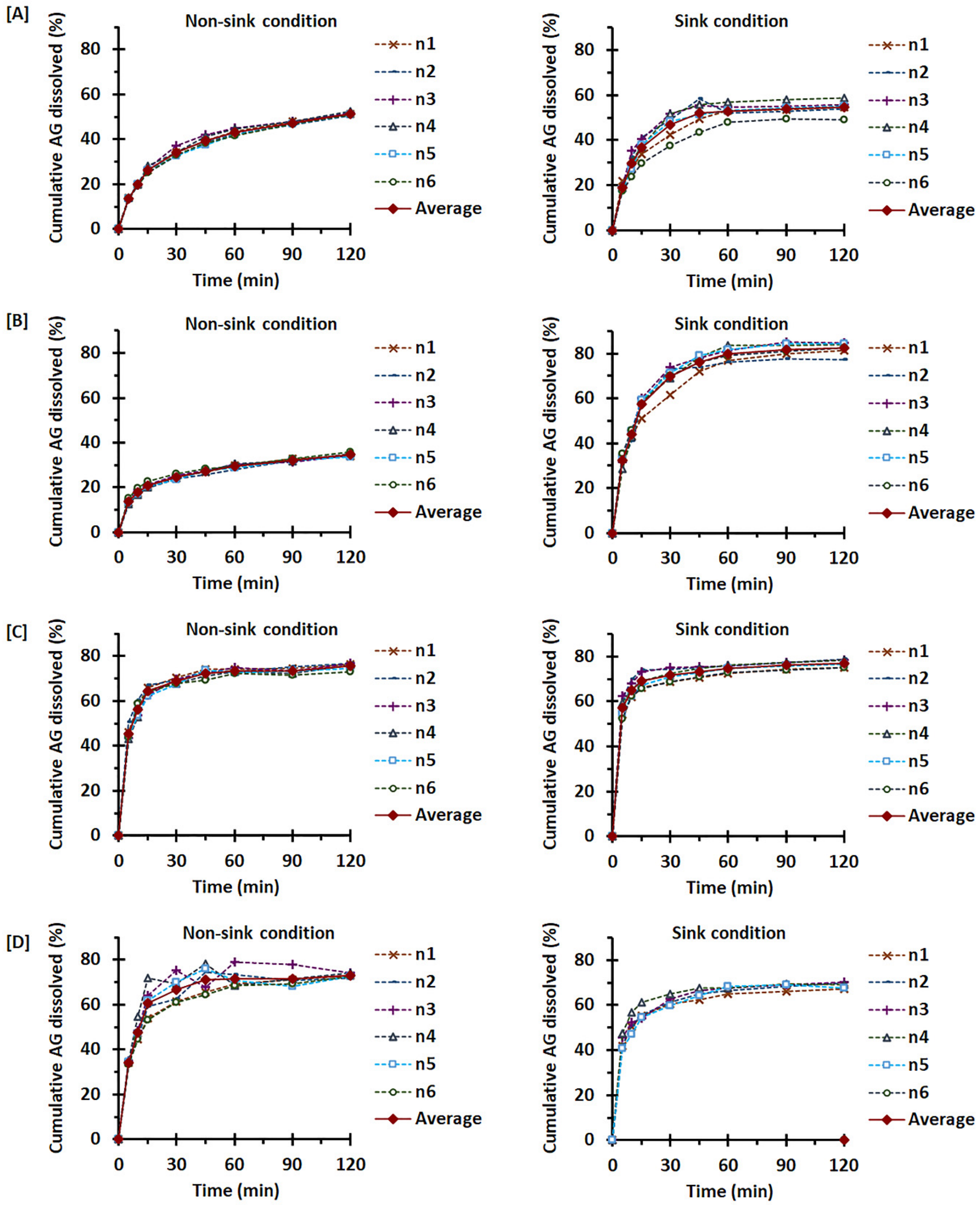

Figure S4. Dissolution of AG molecules under non-sink (left) and sink (right) conditions of pAG–PM [A], APE–S1–PM [B], APE–S2–PM [C], and APE–S3–PM [D].

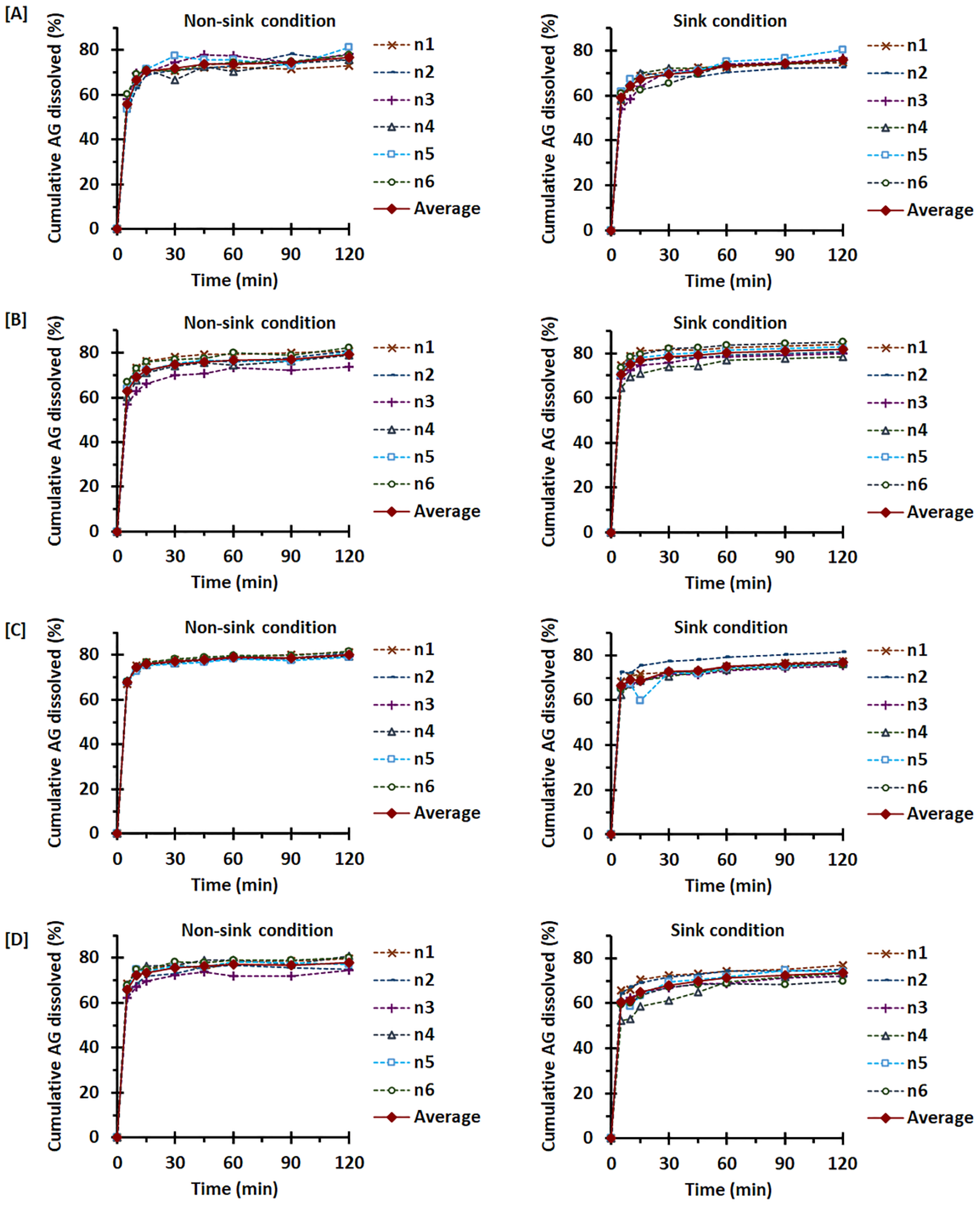

Figure S5. Dissolution of AG molecules under non-sink (left) and sink (right) conditions of pAG–LS [A], APE–S1–LS [B], APE–S2–LS [C], and APE–S3–LS [D].

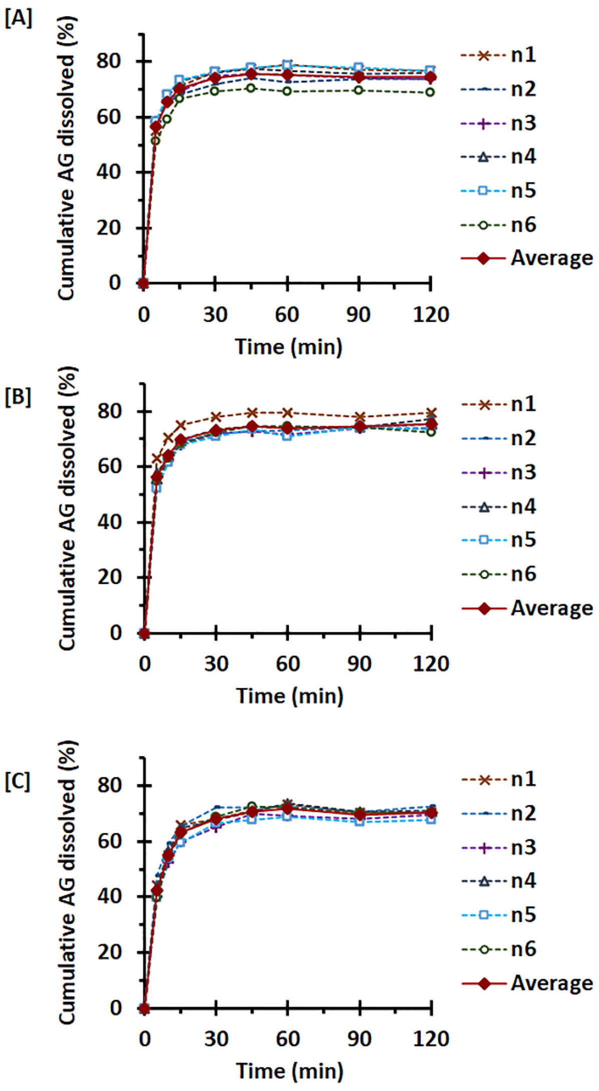

Figure S6. Dissolution of AG molecules under non-sink conditions of 3-month-aged pAG–LS storage under three distinct conditions: 5 ± 3°C (refrigerated) [A], 30°C/75% RH (long-term) [B], and 40°C/75% RH (accelerated) [C].

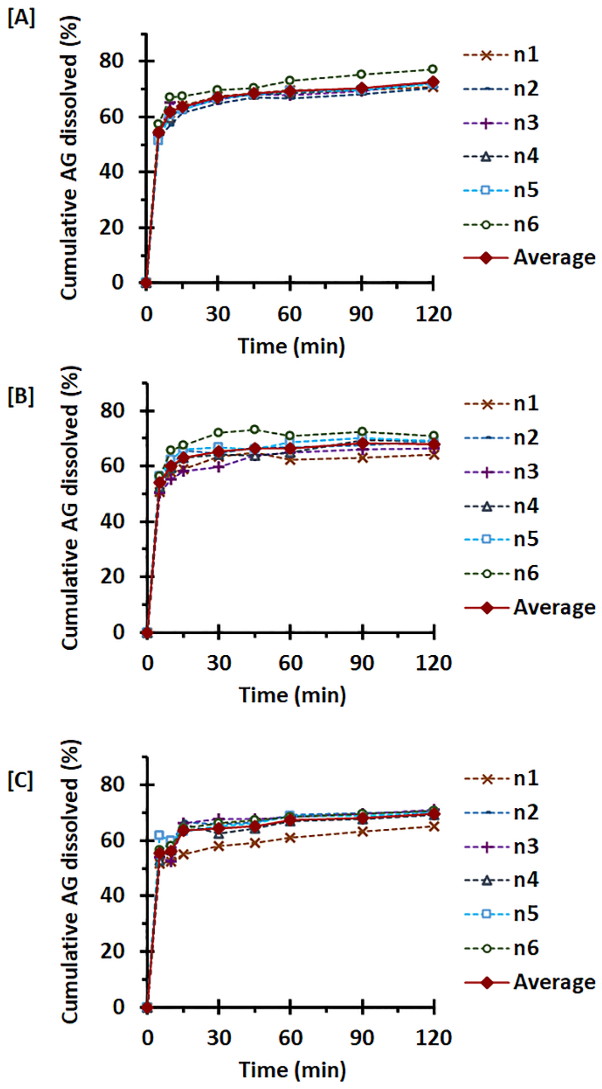

Figure S7. Dissolution of AG molecules under non-sink conditions of 3-month-aged APE–S1–LS storage under three distinct conditions: 5 ± 3°C (refrigerated) [A], 30°C/75% RH (long-term) [B], and 40°C/75% RH (accelerated) [C].

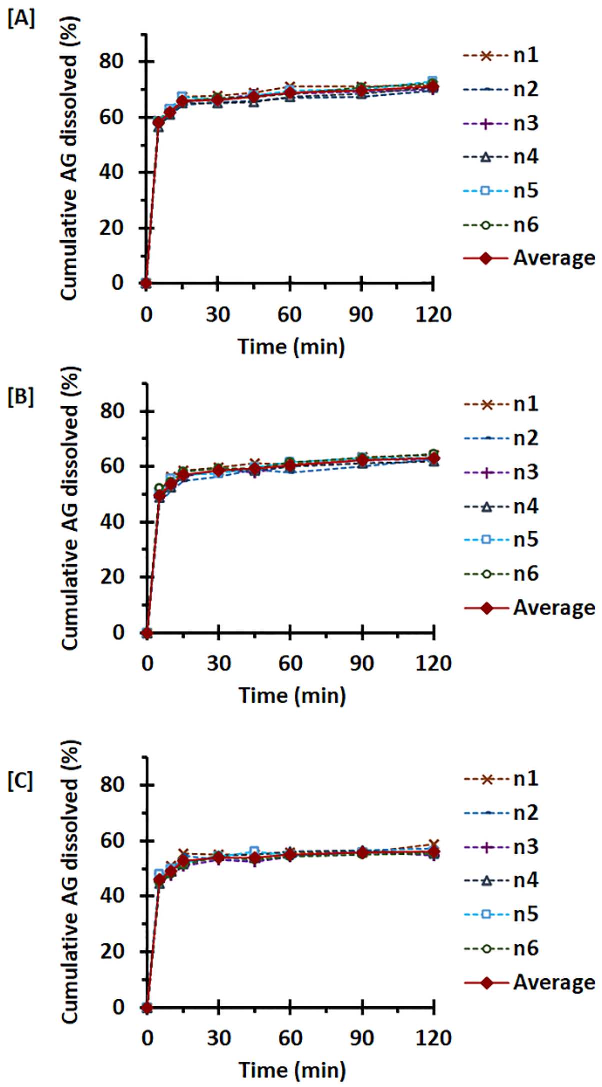

Figure S8. Dissolution of AG molecules under non-sink conditions of 3-month-aged APE–S2–LS storage under three distinct conditions: 5 ± 3°C (refrigerated) [A], 30°C/75% RH (long-term) [B], and 40°C/75% RH (accelerated) [C].

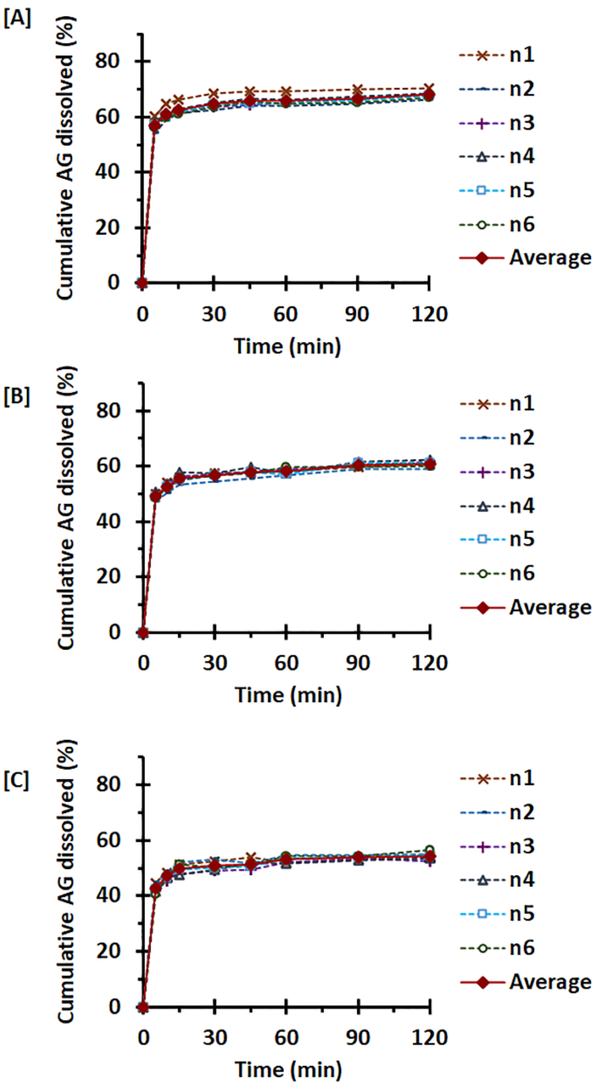

Figure S9. Dissolution of AG molecules under non-sink conditions of 3-month-aged APE–S3–LS storage under three distinct conditions: 5 ± 3°C (refrigerated) [A], 30°C/75% RH (long-term) [B], and 40°C/75% RH (accelerated) [C].
